# Stomatal anatomy and gas exchange dynamics in the *Brachypodium distachyon* complex

**DOI:** 10.1186/s12870-025-07312-0

**Published:** 2025-10-10

**Authors:** Lea S. Berg, Alexander Betekhtin, Anna Milewska-Hendel, Michael T. Raissig

**Affiliations:** 1 Institute of Plant Sciences, University of Bern, Altenbergrain 21, 3013 Bern, Switzerland; 2 Institute of Biology, Biotechnology and Environmental Protection, Faculty of Natural Sciences, University of Silesia in Katowice, 28 Jagiellonska St, 40-032 Katowice, Poland; 3 Oeschger Centre for Climate Change Research, University of Bern, Hochschulstrasse 4, 3012 Bern, Switzerland

**Keywords:** *Brachypodium distachyon* complex, Stomata, Anatomy, Gas exchange, Cell wall, *Brachypodium stacei*, *Brachypodium hybridum*, Ploidy

## Abstract

Stomata, the epidermal pores enabling gas exchange and transpiration, are crucial for plant fitness and global water and carbon cycling. Grasses possess efficient four-celled, graminoid stomatal complexes, where dumbbell-shaped guard cells (GCs) and lateral subsidiary cells (SCs) enable rapid stomatal movements. Yet, how stomatal anatomical properties, such as size and density, affect stomatal gas exchange capacity and kinetics depends on species and growth conditions. Here, we used the *Brachypodium distachyon* complex, a trio of wild grass species consisting of diploid *B. distachyon* (genotype Bd21-3, 2n = 10), diploid *B. stacei* (genotype ABR114, 2n = 20), and their allotetraploid *B. hybridum* (genotype ABR113, 2n = 30)*,* which arose from hybridisation between the two diploids, to integrate comparative stomatal anatomy, physiology, and cell biology and dissect the structure–function relationship of *Brachypodium* stomata. The tetraploid *B. hybridum* formed larger stomata with reduced density, while diploids both featured similarly small stomata yet higher densities. Steady-state gas exchange measurements reflected anatomical predictions in *B. distachyon* and *B. hybridum. B. stacei,* however, severely underperformed and showed significantly lower gas exchange capacity. Ultrastructural and cell wall composition analyses largely revealed conserved GC anatomy and cell wall properties across species. Critically, LM6—targeting pectic (1,5)-α-L-arabinans—showed differential SC wall localisation with signal in *B. distachyon* and *B. hybridum* but absence in *B. stacei*. We propose that altered arabinan distribution could modify SC wall biomechanics, potentially impairing their ability to accommodate GC movement and thus stomatal opening in *B. stacei*. While arabinans are implicated in stomatal flexibility across species, other factors (e.g., ecological niche, mesophyll photosynthesis, signalling, or unassessed wall components) may also contribute to *B. stacei*'s reduced gas exchange.

**Supplementary Information:**

The online version contains supplementary material available at 10.1186/s12870-025-07312-0.

## Introduction

The successful expansion of plants to terrestrial habitats required an outermost cell layer (epidermis) in aerial organs with two major functions: first, a barrier against biotic and abiotic agents and an active interface controlling plant-atmosphere gas exchange [1, 2]. To adopt these functions, plants developed a range of characteristics for leaf epidermal cells; all epidermal cells form a cuticle to efficiently seal the leaves and prevent desiccation, while specialised epidermal cells like guard cells (GCs) in stomatal complexes and trichomes enable gas exchange and fend off herbivores, respectively.

Plant stomata are tiny “breathing” pores on the leaf surface that take up carbon dioxide (CO_2_), which, with the help of sunlight, is turned into sugars and oxygen. At the same time, stomata release water vapour, which is essential for transpiration and root-to-shoot water transport, but also needs to be tightly regulated to conserve water. The water that transpires through stomata contributes 50% of all the water that evaporates from our planet [3]. It is estimated that stomata cycle twice the atmospheric water content per year and enable terrestrial plants to fix 20% of the atmospheric CO_2_ annually [4].

Stomatal cells are among the few plant cells that move without growth by careful and quick adjustment of osmotic potential and dynamic pressurisation of stomatal cells [5]. Therefore, stomatal cell walls must be highly specialised and anisotropically organised to enable directed cell movement and constantly withstand high turgor pressure [6–10]. The GCs are anisotropic due to differential thickening, the orientation of cellulose microfibrils, and polarised localisation of matrix material like pectins, hemicelluloses, lignin, and structural proteins [10–13]. A recent modelling paper showed that the centrally thickened GC walls are crucial for proper stomatal opening and closing in grasses [14], yet we still lack an understanding of the composition of the cell wall that allows them to undergo repeated swelling and deflation in wild grasses like the *B. distachyon* species complex.

Grasses like the model system *B. distachyon* and the cereal crops rice, maize and wheat make four-celled, graminoid stomatal complexes by adding two lateral subsidiary cells (SCs; or “helper cells”) to the two central, dumbbell-shaped GCs [15–17]. The SCs and dumbbell morphology of GCs are developmental innovations that allow grass stomata to open and close more efficiently, thereby contributing to a higher photosynthetic water-use efficiency [14, 18–20].

Previous work has shown that the physiology of plant leaves is influenced by the density and the anatomy of stomata, both intra- and interspecifically [2, 21–29]. There is, however, no clear agreement in the literature on how exactly these parameters influence each other. Stomatal density, for example, was shown to be positively correlated with higher intrinsic water-use efficiency (iWUE) in *Brachypodium distachyon* [21], whereas decreased stomatal density in three grass crops overexpressing a stomatal lineage inhibitor was also shown to positively influence iWUE [26–28].

**Fig. 1:**
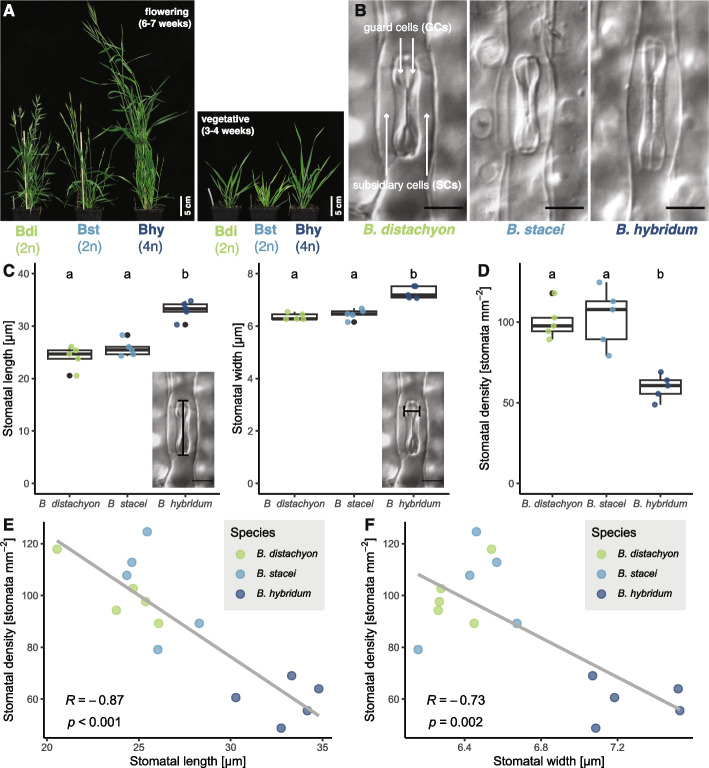
Stomatal anatomy in the *Brachypodium distachyon* species complex strongly correlates with ploidy. **A** The three species *B. distachyon*, *B. stacei* and *B. hybridum* during flowering (6–7 weeks; left panel) and during vegetative growth (3–4 weeks; right panel). Ploidies are indicated. **B** Differential interference contrast image of a single graminoid stomatal complex in the three species as indicated. Dumbbell-shaped GCs and lateral SCs are indicated in the leftmost panel. Scale bar, 10 µm. **C** Stomatal length (left panel) and stomatal width (right panel) of all three species. Insets (same image as in B) depict how length and width were measured. Letters indicate significance (1-way ANOVA, post-hoc Tukey); n = 5 biological replicates and 446–491 stomata per species. **D** Stomatal density in the three species as indicated. Letters indicate significance (1-way ANOVA, post-hoc Tukey); *n* = 5 biological replicates and 177–305 stomata per species. **E**, **F** Stomatal density is negatively correlated with stomatal length (**E**) and stomatal width (**F**). R values and p values of linear regressions are indicated

To compare anatomical and physiological parameters of stomata between different yet closely related species, we used a trio of annuals, sometimes also described as the*’Brachypodium distachyon* species complex’. It consists of the allotetraploid *B. hybridum* (2n = 30) and its diploid progenitors *B. distachyon* (2n = 10) and *B. stacei* (2n = 20) (Fig. 1A) [30, 31]. Importantly, polyploids tend to show an increase in stomatal size (SS) and, concomitantly, a decrease in stomatal number per unit area (= stomatal density (SD), [32, 33]), which allows us to compare distinct stomatal anatomies and their effect on stomatal gas exchange physiology in closely related species.

Here, we comparatively analysed stomatal anatomy in the three annual, wild grasses *B. distachyon* (2n), *B. stacei* (2n) and *B. hybridum* (4n). We calculated the theoretical gas exchange capacity and measured steady-state and dynamic gas exchange responses. Stomatal anatomical parameters nicely explained the divergent steady-state gas exchange in *B. distachyon* and *B. hybridum,* but failed to explain the low gas exchange levels in *B. stacei*. We then examined GC ultrastructure using transmission electron microscopy (TEM) and stomatal cell wall compositions using immunostainings. While ultrastructure seemed conserved, we found that a specific pectin component is absent in SCs of *B. stacei*. We propose that *B. stacei’s* low gas exchange capacity might be linked to its different SC wall properties in addition to its rather unusual ecological niche for this Mediterranean grass family.

## Materials and methods

### Plant material and growth conditions

The plant materials used in this study comply with relevant institutional, national, and international guidelines and legislation. Seeds of *B. distachyon* (2n = 10, accession Bd21-3; origin: Iraq; source: US Department of Agriculture, National Plant Germplasm System, Beltsville, MD, USA), *B. stacei* (2n = 20, accession ABR114; origin: Spain; source: Institute of Biological, Environmental and Rural Sciences, Aberystwyth University, Aberystwyth, UK), and *B. hybridum* (2n = 30, accession ABR113; origin: Portugal; source: Institute of Biological, Environmental and Rural Sciences, Aberystwyth University, Aberystwyth, UK) originated from collections of *Brachypodium* species in Spain and the UK.

Plants were grown in a greenhouse or climate chamber with 18 h light: 6 h dark, average day temperature = 28 °C, average night temperature = 25 °C and average relative humidity = 40%. Prior to planting, seeds were vernalized for 2 days in water at 4 °C with no light exposure.

### Microscopical analysis of stomatal anatomical traits

For microscopical analysis of stomatal complexes, samples were prepared as described previously [21]. Leaves of a subset of individuals (n = 5) were collected after LI-6800 Portable Photosynthesis System with the 6800-01A Multiphase Flash Fluorometer chamber (LI-COR Biosciences Inc., Lincoln, NE, USA) measurements and fixed in 7:1 ethanol:acetic acid. Then, samples were rinsed in water and mounted on slides in Hoyer’s solution. The abaxial side was imaged using a Leica DM2000 DIC microscope. For stomatal density (SD), 3 fields of view (image size 0.198 mm^2^, 20X objective) per leaf were counted, resulting in 29–74 stomata per individual and divided by the image size to obtain stomatal density per mm^2^. For stomatal length (SL) and width at the apices (WA), 73–100 stomata (image size 0.049mm^2^, 40X objective) per individual were measured. Image analysis was done in Fiji (ImageJ 1.53c, [34]). Further analysis of the data was conducted in R (version 4.3.1, [35]) and R Studio (version 2023.06.1). Anatomical *g*_S_max was calculated using the equation optimised for *Brachypodium distachyon* described in [21]. Stomatal density was tested for correlation (two-sided, Pearson test) to stomatal length and width using the smplot2 package (version 0.1.0, [36]). ANOVA (stats package version 4.4.3, [35]) and Tukey’s HSD test (agricolae package version 1.3–6, [37]) were used to check for significant differences between the three species. The following packages were used for file management and final visualisation in R: readxl (version 1.4.3, [38]), tidyverse (version 2.0.0, [39]), ggpubr (version 0.6.0, [40], ggtext (version 0.1.2, [41]) and MetBrewer (version 0.2.0, [42]).

### Leaf-level gas exchange measurements

Physiological parameters for each species were obtained from 3–4 week-old soil-grown, non-flowering plants. The youngest, fully expanded leaf was measured using the LI-6800 Portable Photosynthesis System with the 6800-01A Multiphase Flash Fluorometer chamber (LI-COR Biosciences Inc., Lincoln, NE, USA) in the 2 cm^2^ chamber. Environmental conditions were programmed as described in [21]: flow rate, 500 µmol s^−1^; fan speed, 10,000 rpm and leaf temperature, 28 °C. Gas exchange in reaction to changing light intensities was measured at relative humidity, 40% and CO_2_ concentration, 400 µmol mol^−1^. Values were automatically logged every 60 s with 20-min steps for the light intensities in units of photosynthetically active radiation (PAR), 1000 (high light)—100 (low light)—1000—0 (darkness) PAR m^−2^ s^−1^. PAR is equivalent to Photosynthetic photon flux density (PPFD), which is in μmol m^−2^ s^−1^.

As *Brachypodium* leaves are not broad enough to fill the 2 cm^2^ chamber, data from each run were corrected manually in the Excel sheet by individual leaf area, which was calculated as leaf width multiplied by the chamber diameter. The LI-6800 output already includes values for absolute stomatal conductance (*g*_SW_) and carbon assimilation (*A*), and intrinsic water-use efficiency (iWUE) was calculated as the quotient of *A* divided by *g*_SW_ at a given time point. Steady-state values per parameter were obtained by averaging measurements of 5 min towards the end of each light transition step. To compare stomatal speed between species, lag times (λ), the time constants for stomatal opening (*k*_i_) and closing (*k*_d_), and the maximum slope of *g*_SW_ over time of the fitted curves (*Sl*_max_) were determined according to the model for *g*_SW_ over time described in [43, 44]. These calculations, subsequent averaging of values across all individuals per species, outlier removal, correction by stomatal density and final visualization was conducted in R Studio (R version 4.3.1, [35] and R Studio version 2023.06.01, [45]) using the licornetics R package (version 2.1.1, [46]) that is available on Github with further documentation (https://github.com/lbmountain/licornetics).

### Transmission electron microscopy

For the TEM analysis, leaves of three *Brachypodium* species (control leaves with closed stomata and leaves treated with fusicoccin for the imaging of open stomata), were fixed in 4% glutaraldehyde (GA) and 1% paraformaldehyde (PFA) in 0.1 M phosphate buffered saline (PBS, pH 7.2) (Sigma) and kept for 24 h at 4 °C. After rinsing with PBS (3 × 15 min), material was postfixed in 1% osmium tetraoxide (Serva) in PBS at 4 °C overnight, washed with PBS (3 × 15 min), dehydrated in a graded series of ethanol and gradually embedded in Epon resin (Polysciences). The Epon was polymerised for 24 h at 58 °C. Ultrathin, surface longitudinal-section through the epidermis, 70 nm thick, were obtained using a Leica EM UC6 ultramicrotome and were collected onto carbon-coated copper grids (150 mesh, Electron Microscopy Science). Next, samples were stained with a saturated solution of uranyl acetate (Polysciences) in 50% ethanol for 15 min and 0.04% lead citrate agents (Sigma) for 10 min. The samples were analysed using: Ultra-high resolution field emission scanning electron microscope UHR FE-SEM Hitachi SU8010 (Hitachi High-Technologies Corporation, Tokyo, Japan); 2/Jeol JEM-3010 (300 kV) HRTEM equipped with an EDS (Energy Dispersive Spectrometry) spectrometer and a Gatan 2 k × 2 k Orius TM 833 SC200D CCD camera (photos from supplementary material).

### Immunohistochemical analysis

For immunohistochemical analysis, samples were prepared as described earlier with some modifications [47]. In brief, small pieces of leaves (about 3 × 4 mm in size) of three *Brachypodium* species, were fixed in 3% paraformaldehyde (PFA) and 1% glutaraldehyde (GA) in 0.1 M phosphate buffered saline (PBS, pH 7.2; Sigma-Aldrich, St. Louis, MO, USA) and kept overnight at 4 °C. Subsequently, the samples were washed in PBS, followed by a dehydration in increasing ethanol concentrations and then gradually embedded in LR White resin (Polysciences, Warrington, PA). During embedding, the abaxial sides of the leaves were placed at the bottom of the embedding capsules. After polymerisation at 57 °C (24 h), the samples were cut near the surface around the embedded beam, parallel to the abaxial surface of the leaf. Sections were cut into 1 µm thick longitudinal sections and were placed on glass slides coated with poly-L-lysine (Polysine®, Menzel Thermo Scientific, Jiangsu, China). Next, the sections were treated with blocking buffer (BB; 2% foetal calf serum and 2% bovine serum albumin in PBS) for 30 min at RT to block nonspecific binding sites. Subsequently, the samples were incubated with primary monoclonal antibodies (Plant Probes, Leeds, UK; Table 1) diluted 1:20 in a BB and kept at 4 °C overnight. Following the washing with BB (3 × 10 min), sections were incubated with the secondary antibody, labelled with AlexaFluor 488 goat anti-rat IgG (Jackson ImmunoResearch Laboratories, Cambridgeshire, UK) diluted 1:100 in the BB and kept at RT for 2 h. The sections were rinsed in the BB (3 × 10 min), stained with 0.01% (w/v) fluorescent brightener 28 (FB28; Sigma-Aldrich, St. Louis, MO, USA) diluted in PBS. Next, the samples were washed in PBS (3 × 10 min) and distilled water (5 × 5 min) and mounted with the anti-fading medium Fluoromount (Sigma-Aldrich, St. Louis, MO, USA). To confirm the specificity of the secondary antibody, negative controls were prepared by substituting the primary antibody step with BB.

**Table 1:** List of the monoclonal primary antibodies used in the study, the epitopes they recognised, and references. GlcA—glucuronic acid, HG—homogalacturonan, RG I – rhamnogalacturonan I

Antibody	Recognised epitope	References
*Pectins*		
LM6	linear pentasaccharide in (1 → 5)-α-L-arabinans (RG I side chain); also labels arabinogalactan proteins	Willats et al., 1998 [61]
JIM5	HG domain of pectic polysaccharides, recognises partially methyl-esterified epitopes of HG, can also bind to unesterified HG	Knox et al., 1990 [62]
JIM7	HG domain of pectic polysaccharides, recognises partially methyl-esterified epitopes of HG but does not bind to unesterified HG	Knox et al., 1990 [62]
LM13	Linear (1–5)-α-l-arabinan (RG I)	Moller et al., 2008 [63]; Verhertbruggen et al., 2009 [64]
2F4	non-esterified or de-esterified HG that are cross-linked by calcium	Liners et al., 1989 [65]
*AGPs*		
LM2	Arabinogalactan/Arabinogalactan protein, carbohydrate epitope containing β - linked GlcA	Smallwood et al. 1996 [66]
*Hemicelluloses*		
LM25	XLLG, XXLG and XXXG motifs of xyloglucan	Pedersen et al., 2012 [67]

For whole-mount immunostaining, samples were prepared as described earlier with some modifications [48]. In brief, small pilled pieces of leaves epidermis layer of three *Brachypodium* species, were fixed in 3% PFA and 1% GA in 0.1 M PBS (pH 7.2; Sigma-Aldrich, St. Louis, MO, USA) and kept overnight at 4 °C. Subsequently, the samples were washed in PBS, treated with methanol for cuticle solubilisation and 0.2% of driselase for partial digestion of the cell wall which help for easier penetration of the antibodies. Then pieces of the epidermis layer were treated with membrane permeabilisation solution, which contained 3% IGEPAL C630, 10% DMSO in 0.1 M PBS. Next, the samples were treated with blocking buffer (BB; 2% foetal calf serum and 2% bovine serum albumin in PBS) for 2 h at RT to block nonspecific binding sites. Subsequently, the samples were incubated with primary monoclonal antibodies (Plant Probes, Leeds, UK; Table 1) diluted 1:20 in a BB and kept at 4 °C overnight. Following the washing with BB (3 × 10 min), the samples were incubated with the secondary antibody, labelled with AlexaFluor 488 goat anti-rat IgG (Jackson ImmunoResearch Laboratories, Cambridgeshire, UK), diluted 1:100 in the BB and kept at 37 °C for 2 h. The samples were rinsed in the BB (3 × 10 min), stained with 0.01% (w/v) fluorescent brightener 28 (FB28; Sigma-Aldrich, St. Louis, MO, USA) diluted in PBS. Next, the samples were washed in PBS (3 × 10 min) and distilled water (5 × 5 min) and mounted with the anti-fading medium Fluoromount (Sigma-Aldrich, St. Louis, MO, USA). To confirm the specificity of a secondary antibody, negative controls were made by applying the BB instead of the primary antibody step. Images were acquired using a Leica SP8 confocal microscope. Each section was captured in two channels: Alexa Fluor 488 (excitation peak at 488 nm) and CF (excitation peak at 360 nm). Image processing was performed using ImageJ (Wayne Rasband, National Institutes of Health, USA) or Imaris (Bitplane) software.

## Results

### Stomatal anatomy in the *Brachypodium* species complex

Polyploid organisms form bigger stomata than diploid species [31, 33]. To compare stomatal anatomy between the allotetraploid *Brachypodium hybridum* and its diploid progenitor species, *Brachypodium distachyon* and *Brachypodium stacei*, we imaged fixed and cleared mature leaves from 3 to 4-week-old individuals of all three species (Fig. 1A, B). We measured stomatal length and width (Fig. 1C) and determined abaxial stomatal density (Fig. 1D) for all three species.

While *B. distachyon* and *B. stacei* stomata showed similar stomatal length and width, the polyploid *B. hybridum* showed significantly longer and wider stomata (Fig. 1B, C; Table 2). Furthermore, *B. hybridum* had significantly fewer (abaxial) stomata per area, with around 60 stomata per mm^2^, whereas *B. distachyon* and *B. stacei* had similar levels of around 100 stomata per mm^2^ (Fig. 1D, Table 2). As shown previously for other plants across many taxa, stomatal density was negatively correlated with stomatal size among these three species (Fig. 1E, F).

**Table 2:** Stomatal gas exchange and anatomical parameters of the *Brachypodium distachyon* species complex

		***B. distachyon***	***B. stacei***	***B. hybridum***
*g*_SW_ [mol m^−2^ s^−1^]	High light 1	0.273 ± 0.000	0.170 ± 0.002	0.304 ± 0.000
	Low light	0.082 ± 0.001	0.069 ± 0.000	0.097 ± 0.001
	High light 2	0.252 ± 0.001	0.163 ± 0.001	0.261 ± 0.003
	Darkness	0.018 ± 0.001	0.043 ± 0.001	0.032 ± 0.001
*A* [µmol m^−2^ s^−1^]	High light 1	18.66 ± 0.18	7.09 ± 0.44	18.45 ± 0.04
	Low light	2.93 ± 0.11	1.45 ± 0.06	3.21 ± 0.09
	High light 2	18.39 ± 0.42	7.63 ± 0.69	17.55 ± 0.10
iWUE [µmol (C) mol (H_2_O)^−1^]	High light 1	69.89 ± 0.74	40.91 ± 3.06	61.58 ± 0.13
	Low light	36.04 ± 1.54	20.52 ± 1.27	34.38 ± 0.71
	High light 2	74.21 ± 1.72	46.52 ± 4.44	70.15 ± 0.67
Anatomical *g*_S_max [mol m^−2^ s^−1^] kinetics leaves		0.378 ± 0.025	0.422 ± 0.061	0.333 ± 0.051
*g*_SW_ (high light 2)/Anatom. *g*_S_max [%]		67	39	78
Stomatal density [mm^−2^]		100.3 ± 17.9	102.7 ± 26.5	59.6 ± 11.5
Stomatal length [µm]		24.1 ± 2.8	25.8 ± 2.9	33.1 ± 3.2
Stomatal width [µm]		6.4 ± 0.6	6.5 ± 0.6	7.3 ± 0.6
Stomatal length/width ratio		3.8 ± 0.5	4.0 ± 0.6	4.6 ± 0.5

**Fig. 2:**
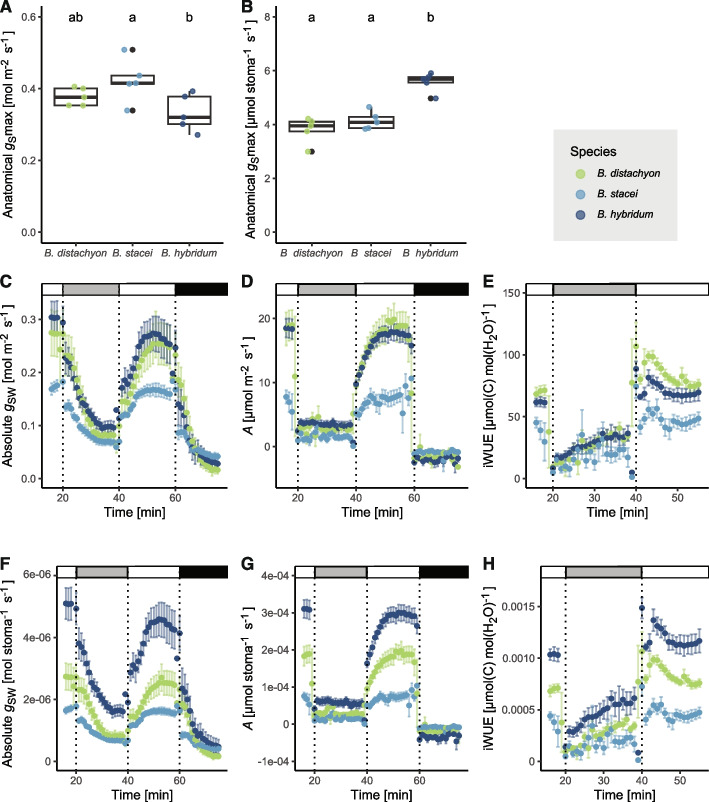
Differential stomatal gas exchange capacity and kinetics in the diploid species despite highly similar stomatal anatomy. **A**, **B** Theoretical stomatal gas exchange capacity (anatomical *g*_s_max) per leaf area (A) or per stoma (B) calculated according to [21]. Letters indicate significance (1-way ANOVA, post-hoc Tukey); *n* = 5 biological replicates. **C**-**E** Dynamic gas exchange measurements in response to changing light conditions (1000 to 100 to 1000 to 0 PAR). Shown are absolute stomatal conductance (*g*_sw_; C), carbon assimilation (*A*; D), and intrinsic water-use efficiency (iWUE; E) per leaf area. Colours indicate species, dashed vertical lines indicate light intensity changes; *n* = 5 individuals. **F**–**H** Dynamic gas exchange measurements in response to changing light conditions normalised per stoma (1000 to 100 to 1000 to 0 PAR). Shown are absolute stomatal conductance (*g*_sw_; F), carbon assimilation (*A*; G), and intrinsic water-use efficiency (iWUE; H) normalised per stoma. Colours indicate species, dashed vertical lines indicate light intensity changes; n = 5 individuals

We then calculated the theoretical maximum stomatal conductance capacity (anatomical *g*_S_max) based on the measured anatomical parameters for the three species [21]. Anatomical *g*_s_max between the three species was highly comparable, even though there was a significant difference between *B. stacei* and *B. hybridum* (Fig. 2A, Table 2). This suggested that a negative correlation between size and density was sufficient to buffer leaf-level gas exchange capacity. Indeed, calculating anatomical *g*_s_max per stoma rather than per leaf area showed no difference between the diploids but significantly higher values for *B. hybridum* (Fig. 2B, Table 3).

### Licornetics, an R package to easily plot and analyse gas exchange data

Our anatomical *g*_S_max values suggested that in vivo, leaf-level gas exchange measurements would also show similar gas exchange capacity among the three species. We, therefore, conducted leaf-level gas exchange measurements in response to changing light intensities for the tetraploid *B. hybridum* and its diploid progenitors *B. distachyon* and *B. stacei*.

To facilitate the calculation and visualisation of plant gas exchange parameters and stomatal speed kinetics, we developed the R package licornetics (https://github.com/lbmountain/licornetics) that includes two functions. The first function is licorplots, and it uses the raw infrared gas analyser Excel files as input and visualises various parameters such as absolute or relative (i.e. normalised) *g*_SW_, *A*, and iWUE over time (examples see Fig. 2C-H). It can also plot other measurements obtained from the system, like internal CO_2_ concentration (C_i_), atmospheric CO_2_ concentration (C_a_), transpiration (*E*), etc. Second, the licorvalues function uses the same input data to calculate steady-state gas exchange values for *g*_SW_, *A* and iWUE and summarises these values in a table (e.g. Table 2). In addition, it models stomatal kinetics and calculates the speed parameters according to [43, 44]. Speed parameters are also summarised in a table (e.g. Table 4), and the model fits per individual or identifier group (i.e. genotype or species) are plotted alongside (Fig. S1).

### Gas exchange physiology in the *Brachypodium* species complex

We then used licornetics to comparatively plot and analyse the gas exchange data collected for all three *Brachypodium* species. In darkness (0 photosynthetically active radiation (PAR)) and low light (100 PAR), the three species displayed similar levels of steady-state stomatal conductance (*g*_SW_) and carbon assimilation (*A*) (Fig. 2C, D; Table 2). Under high-light conditions (1000 PAR), however, *B. distachyon* and *B. hybridum* reach comparable levels of *g*_SW_ and *A,* whereas *B. stacei* reaches only around 60% of the *g*_SW_ and about 40% of the *A* levels compared to the other species (Fig. 2C-D; Table 2). To obtain intrinsic water-use efficiency (iWUE) values (i.e. water lost per carbon fixed during photosynthesis), we divided *g*_SW_ by *A*. Both *B. hybridum* and *B. distachyon* reached iWUE levels of around 70 µmol carbon per mol water in high light, while *B. stacei* remained below 50 µmol carbon per mol water, suggesting its photosynthesis to be less water-use-efficient (Fig. 2E; Table 2). Considering the identical stomatal anatomy between *B. distachyon* and *B. stacei*, the strongly reduced physiological gas exchange capacity of *B. stace*i compared to *B. distachyon* was unexpected.

On the other hand, stomatal gas exchange parameters between *B. distachyon* and *B. hybridum* were very similar, suggesting that differing stomatal sizes and densities balanced each other. Indeed, when we normalised the leaf-level gas exchange values by stomatal density to obtain gas exchange values per stoma (Fig. 2 F–H, Table 3), we found that a single *B. hybridum* stoma performed best for *g*_SW_, *A* and iWUE in all light conditions. *B. stacei,* however, still underperformed significantly (Fig. 2F-H, Table 3), further indicating that the underperformance cannot be explained by stomatal anatomy alone. Finally, operational opening at high light (*g*_SW_ at high light divided by anatomical *g*_S_max) of *B. distachyon* was 67%, for *B. hybridum* 78%, but for *B. stacei* only 39% of the theoretically calculated maximum opening capacity (Tables 2 and 3).

**Table 3:** Stomatal gas exchange parameters normalised per stoma of the *Brachypodium distachyon* species complex

		***B. distachyon***	***B. stacei***	***B. hybridum***
*g*_SW_ [µmol Stoma^−1^ s^−1^]	High light 1	2.72 ± 0.00	1.65 ± 0.02	5.10 ± 0.01
	Low light	0.82 ± 0.01	0.67 ± 0.00	1.62 ± 0.01
	High light 2	2.51 ± 0.01	1.59 ± 0.01	4.38 ± 0.06
	Darkness	0.18 ± 0.01	0.42 ± 0.01	0.53 ± 0.02
*A* [pmol Stoma^−1^ s^−1^]	High light 1	186.02 ± 1.78	69.05 ± 4.28	309.62 ± 0.62
	Low light	29.17 ± 1.10	14.08 ± 0.60	53.83 ± 1.54
	High light 2	183.24 ± 4.14	74.32 ± 6.74	294.43 ± 1.68
iWUE [pmol (C) µmol (H_2_O)^−1^]	High light 1	697.00 ± 7.41	398.00 ± 29.80	1033.37 ± 2.23
	Low light	359.00 ± 15.30	200.00 ± 12.30	577.00 ± 11.90
	High light 2	740.00 ± 17.10	453.00 ± 43.30	1177.05 ± 11.30
Anatomical *g*_S_max [µmol Stoma^−1^ s^−1^] kinetics leaves		3.80 ± 0.48	4.14 ± 0.34	5.58 ± 0.36
*g*_SW_ (high light 2)/Anato. *g*_S_max [%]		67	39	78

We then used the stomatal response data to calculate stomatal opening and closing kinetics using a previously generated model (see Licornetics, [43, 44, 46]). The measurements depicted in Fig. 2C-F were split into three light transition zones to consider stomatal closing and opening independently. We calculated lag times (λ), time constants for stomatal opening (*k*_i_) and closing (*k*_d_) and the maximum slope of *g*_SW_ over time (*Sl*_max_) for these transition zones and thereby obtained values for stomatal closing from high light (1000 PAR) to low light (100 PAR), stomatal opening from low light to high light and once more stomatal closing from high light to darkness (0 PAR) (Table 4, Fig. S1). For *k* and λ, *B. hybridum* performed best except for λ in the stomatal closing in the low light transition. Considering the actual maximum speed of opening and closing indicated with the *Sl*_max_ values, *B. stacei* was the slowest in all three transition steps, with about 2–3 times less steep slopes (Table 4, Fig. S1). Interestingly, for *B. distachyon,* stomatal closing was faster than stomatal opening according to all three speed parameters (*k*_i_, *k*_d_, and *Sl*_max_). For the other two species, both *k* and *Sl*_max_ values indicated faster opening than closing (Table 4). Regarding λ, however, all three species reacted more quickly to a light change for closing rather than for opening (Table 4).

**Table 4:** Stomatal gas exchange kinetics in the *Brachypodium distachyon* species complex

Anato.g_s_max		*B. distachyon*	*B. stacei*	*B. hybridum*
*k*_d_ [min^−1^]	High light to low light/ Stomatal closing	1.75 ± 0.27	2.48 ± 0.35	3.29 ± 0.61
λ [min]		1.24 ± 0.53	0.68 ± 0.53	0.66 ± 0.27
Sl _max, closing_ [mmol m^−2^s^−2^]		−0.59 ± 0.10	−0.18 ± 0.03	−0.30 ± 0.07
*k*_i_ [min^−1^]	Low light to high light/Stomatal opening	2.08 ± 0.41	1.42 ± 0.24	1.14 ± 0.20
λ [min]		1.44 ± 0.59	1.97 ± 0.31	2.13 ± 0.54
Sl_max_, _opening_[mmol m^−2^s^−2^]		0.41 ± 0.14	0.22 ± 0.03	0.57 ± 0.17
*k*_d_ [min^−1^]	High light to darkness/Stomatal closing	2.01 ± 0.25	2.04 ± 0.27	1.74 ± 0.38
λ [min]		1.05 ± 0.45	1.13 ± 0.51	0.63 ± 0.45
*Sl*_max,closing_[mmol m^−2^s^−2^]		−0.57 ± 0.11	−0.15 ± 0.05	−0.44 ± 0.10

In summary, larger stomata in the tetraploid *B. hybridum* were in line with higher leaf-level gas exchange values per stoma. Its lower stomatal density, however, compensated for this effect on a per leaf area basis, making its gas exchange performance similar to diploid *B. distachyon* (Table 2, 3). The other diploid species *B. stacei*, which does not differ significantly in stomatal size and density compared to *B. distachyon,* severely underperformed. It was unable to reach the same opening and closing speed or steady-state levels of gas exchange in high-light conditions.

### Ultrastructure of *Brachypodium* stomata

**Fig. 3:**
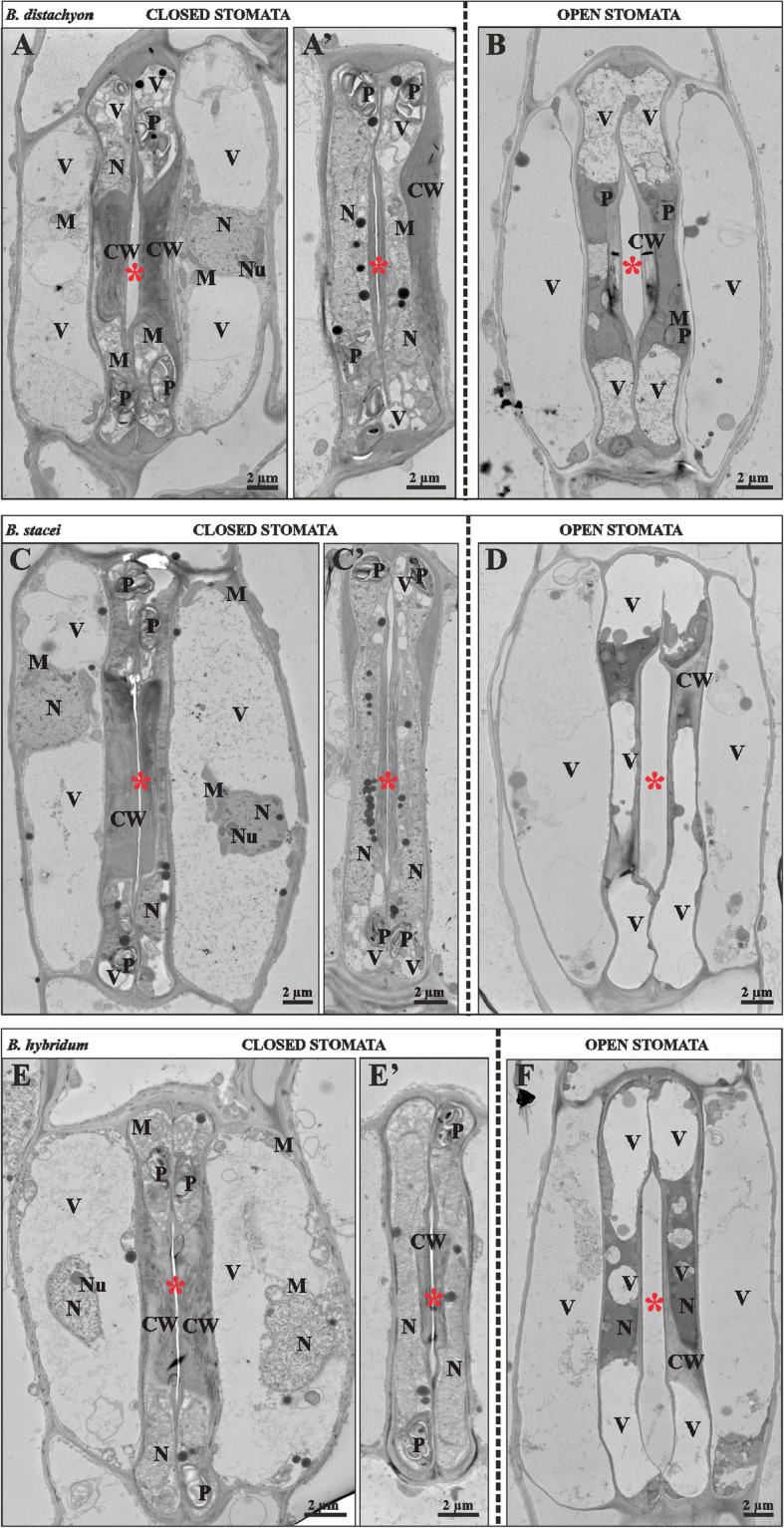
Ultrastructure of closed and open stomata of *B. distachyon* (A, A’ and B)*, B. stacei* (C, C’ and D) and *B. hybridum* (E, E’ and F). CW: cell wall; M: mitochondria; N: nucleus; Nu: nucleolus; P: plastid; V: vacuole; red asterisks: stomatal pore

As our data on stomatal size and density of the three species could not explain the physiological differences we observed, especially between the two diploid species, we also investigated stomatal anatomy on the subcellular level. To observe the subcellular peculiarities of grass stomata in the three species, we performed transmission electron microscopy on periclinal, longitudinal sections through both open (Fusicoccin-treated) and closed (treated with abscisic acid) stomata of *B. distachyon*, *B. stacei* and *B. hybridum* (Fig. 3).

As suggested from 3D images [9, 14], GC wall thickness is highly anisotropic. The thickest electron-dense walls were found near the pore, and the thinnest walls between GC apices and neighbour cells (subsidiary and pavement cells). Vacuoles were only found in the apices, and the large, grass-specific symplastic connections linking both GCs were clearly visible in all three species. The nuclei in the GCs of the closed stomata were elongated and had a dumbbell shape (Fig. 3A', C, C', E'). Moreover, these cells were characterised by dense cytoplasm and the presence of plastids with large starch grains (amyloplasts), numerous mitochondria and small vacuoles (Fig. A, A', C, C', E, E'). The protoplasts of SCs were characterised by the presence of a large vacuole that occupied almost the entire volume of the cell (Fig. 3 A, A, C, C', E, E'). The nuclei in these cells were large and round, and featured mitochondria clusters in the vicinity (Fig. 3 A, C, E). SC walls were very thin (Fig. 3 A, C, E). During stomatal opening, ultrastructural changes in GCs were observed as the volume of the vacuoles increased significantly (Fig. 3B, D, F). Strikingly, some of the vacuoles built a continuous structure connecting the GC apices through symplastic connections, strongly suggesting that grass GCs indeed act as a single unit [49]. The ultrastructure of SCs remained unchanged during stomatal opening (Fig. 3B, D, F).

Apart from the large symplastic connection in apices, plasmodesmata connections were absent between sister GCs (Supplementary S2A, A') and also between GCs and SCs or GCs and the apical and basal interstomatal pavement cell (Supplementary S2 B, B'). However, PD occurred between subsidiary and pavement cells and were branched (Supplementary S2C), twined (Supplementary S2C') or single. Overall, this suggests that the GCs are indeed symplastically isolated from the surrounding cells, yet SCs might not be. This raises the question of how SC turgor can be inversely regulated independently of surrounding pavement cells, which seemed to be a requirement for grass stomatal function [14, 18].

In conclusion, studies using TEM showed that the ultrastructure of closed and open stomata did not differ between the three *Brachypodium* species, but revealed striking differences between stomatal opening and closing, confirmed symplastic GC isolation and corroborate the proposed theory that grass GCs must act as a single unit linked by large symplastic connections in their apices for efficient and coordinated stomatal opening [49, 50].

### Distribution of different cell wall components in *Brachypodium* graminoid stomata

Another possible explanation for the significant differences in opening and closing capacity and kinetics of *B. stacei* could be differences in cell wall composition. To determine the chemistry of the cell wall, a set of monoclonal antibodies against specific cell wall epitopes such as pectins (LM6, 2F4, JIM5, LM13, JIM7), arabinogalactan proteins (AGPs; LM2), and hemicellulose: xyloglycan (LM25) were used (Table 1). We performed immunostainings on both whole-mount samples and histological sections. The immunostaining on the histological sections confirmed that there are little or no changes to stainings in whole-mount samples that may be caused by enzymatic cell wall treatments, as immunocytochemical analysis in the histological section does not include treatment of the samples with cell wall-degrading enzymes (Figure S3-S11). For many of these antibodies, we found both intracellular signals and signals at the cell outlines. We assume that the intracellular signals are the cell wall components being made in endosomal compartments and transported to the apoplast. We, therefore, will discuss below only the signals found at the cell wall and plasmalemma.

**Fig. 4:**
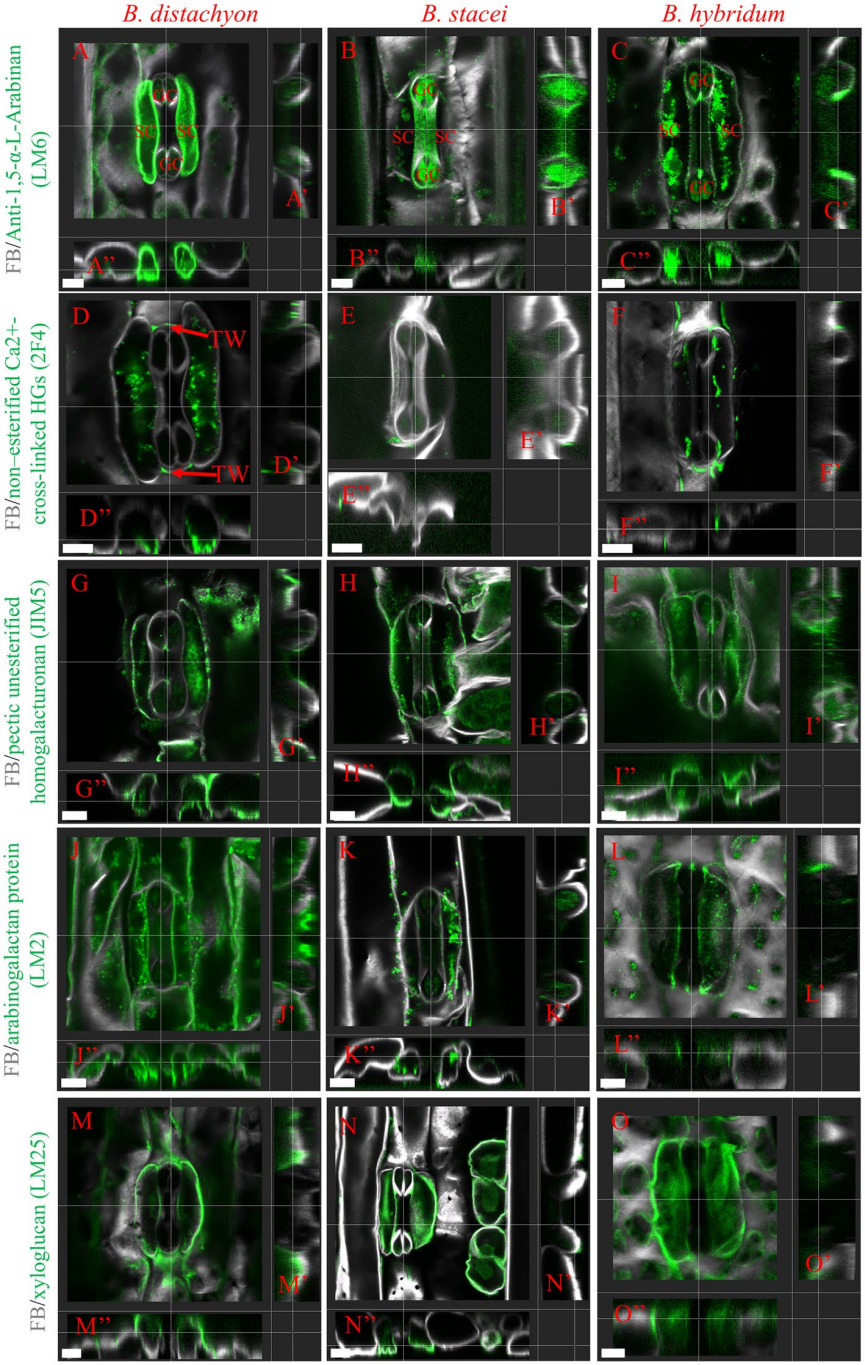
Whole-mount immunolocalization of anti-(1,5)-α-L-arabinan (LM6 epitope) in *B. distachyon* (A–A’’), *B. stacei* (B–B’’), and *B. hybridum* (C–C’’); non-esterified Ca^2^⁺-cross-linked homogalacturonan (2F4 epitope) in *B. distachyon* (D–D’’), *B. stacei* (E–E’’), and *B. hybridum* (F–F’’); unesterified homogalacturonan (JIM5 epitope) in *B. distachyon* (G–G’’), *B. stacei* (H–H’’), and *B. hybridum* (I–I’’); arabinogalactan protein (LM2 epitope) in *B. distachyon* (J–J’’), *B. stacei* (K–K’’), and *B. hybridum* (L–L’’); and xyloglucan (LM25 epitope) in *B. distachyon* (M–M’’), *B. stacei* (N–N’’), and *B. hybridum* (O–O’’). FB—fluorescent brightener. Scale bars 5 μm

The first antibody, LM6, recognises a linear pentasaccharide in pectic (1–5)-α-L-arabinans and may also recognise AGPs. In GCs, LM6 is primarily found in the plasmalemma with a slight preference for the cell walls oriented towards the pore (Fig. 4A-C, Fig. S3A-J; Fig. S4A-B, Video S1A-C). In SCs, however, LM6 signal is found very strongly in the cell walls of *B. distachyon* and weaker in *B. hybridum*, but is largely absent in *B. stacei* SCs. Overall, the signal intensity was different between the analysed species, and was similar between the GCs but not the SCs of the three species.

The monoclonal antibody 2F4 is specific to a particular polygalacturonic acid conformation induced by a given calcium-to-sodium ratio. The strongest signal of this antibody was found at the GCs’ basal and apical transverse wall (TW) at the GC apices (Fig. 4D-F, Fig. S5A-J, Video S2A-C). In the SC cell walls, no cell wall signal was found. Overall, this very polarised signal at transverse walls is conserved among the species.

Next, JIM5 recognises partially methyl-esterified homogalacturonan but can also bind to un-esterified homogalacturonan. JIM5 was localised in the cell wall of both GCs and SCs (Fig. 4G-I, Fig. S6A-J, Video S3A-C). In the GCs, the signal was localised mostly to the apices with a slight tendency towards the pore. Moreover, in SCs, the signal is localised in the distal walls that connect to the neighbouring epidermal cells in all three species. Again, the distribution of JIM5 signals seemed conserved between the three species.

LM2, which recognises a carbohydrate epitope containing β-linked glucuronic acid of AGPs. This antibody was localised to the cell wall of both GCs and SCs, with the strongest signal in the shared GC-SC apoplast for all three species (Fig. 4J-L, Fig. S7A-J, Fig. S8A-J, Video S4A-C). While there was a significant difference in staining intensity between the species, the GC-SC apoplast was stained the strongest for all three (Fig. 4J-L, Fig. S7A-J, Fig. S8A-J).

Finally, LM25, which recognises xyloglucan, was the most abundant in the SCs and GCs of all three species (Fig. 4M-O, Fig. S9A-J, Video S5A-C).

**Fig. 5:**
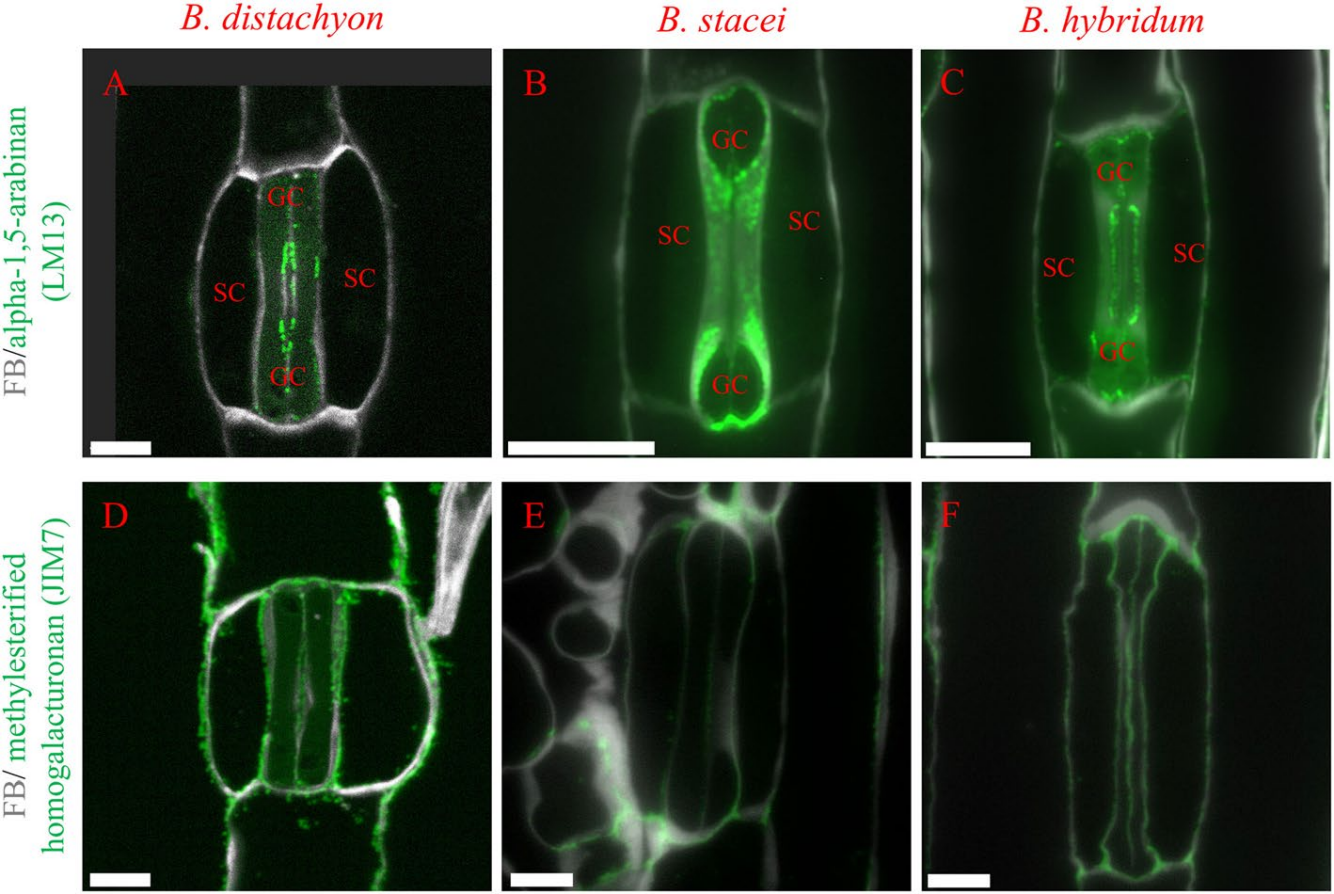
Immunolocalisation on histological sections of (1,5)-α-L-arabinan (LM13 epitope) in *B. distachyon* (**A**), *B. stacei* (**B**), and *B. hybridum* (**C**) and methylesterified homogalacturonan (JIM7 epitope) in *B. distachyon* (**D**), *B. stacei* (**E**), and *B. hybridum* (**F**). Scale bars 5 μm

In the case of LM13 and JIM7, we could not detect a fluorescence signal in whole-mount samples. We could, however, detect signals for both LM13 and JIM7 in histological sections (Fig. 5, Fig. S10-S11). LM13 recognises a linear epitope in (1–5)-α-L-arabinans. This antibody binds to a specific subset of pectic arabinans and longer stretches of 1,5-linked arabinosyl residues. LM13 was present in the plasmalemma and cell wall of GCs and with a very weak signal in the cell wall of SCs, yet no obvious difference was found between the three species (Fig. 5A-C, Fig. S10A-B). Finally, the JIM7 antibody recognises partially methyl-esterified homogalacturonan, but does not bind to un-esterified homogalacturonan. The signal from this antibody was present in the cell wall of both GCs and SCs, with no particular difference found between the three species (Fig. 5D-F, Fig. S11A-B).

Overall, cell wall components seemed to be largely distributed similarly between the three *Brachypodium* species. The only notable exception was LM6, which detects a linear pentasaccharide in pectic (1–5)-α-L-arabinans and may recognise AGPs. This modification was not found in *B. stacei* SCs and might contribute to the reduced opening capacity of *B. stacei* stomata.

## Discussion

Several previous studies have reported effects of stomatal anatomy and density on leaf-level gas exchange parameters both within a single species and between different species, yet the significance and direction of correlations depended on the species context and environmental conditions [2, 21–29]. Here, we compared the allotetraploid C_3_ grass *B. hybridum* and its diploid progenitors *B. distachyon* and *B. stacei*. This species complex allowed us to compare anatomical and physiological traits between closely related species as well as between a polyploid and its diploid parental species. Furthermore, we developed an R package that allows quick and accessible plotting and calculations of dynamic gas exchange measurements (https://github.com/lbmountain/licornetics).

In accordance with a recent study that compared polyploids with their progenitors [33], we confirmed that polyploid *B. hybridum* had larger stomata and lower stomatal density, but still reached similar or slightly higher leaf-level gas exchange parameters than the diploids. Furthermore, we show that on a single stoma level *B. hybridum* has distinctly higher *g*_SW_, *A* and iWUE than the other two species under high light conditions. Thus, longer and wider GC complexes seem to compensate or even overcompensate for lower stomatal density in the polyploid compared to the diploid parental lines.

*Brachypodium* species, like all grasses, form a unique stomatal morphotype, where dumbbell-shaped GCs are flanked by parallel SCs [15, 16]. Such graminoid stomata were shown to enable faster stomatal movements than ellipsoid or kidney-shaped stomata [18, 44]. All three species in this study align with this general hypothesis, and in comparison to a study with 15 species, including 6 other grasses [44], the stomatal kinetics values we obtained are in a similar range with other grasses such as the major crops *Oryza sativa* and *Zea mays* and show faster stomatal movements than eudicots with kidney-shaped stomata. Whether this speed advantage is due to the dumbbell morphology of GCs, the lateral SCs, or both remains to be determined.

We previously found an intraspecific link between stomata anatomy and physiology within *B. distachyon* grown in different seasons [21]. Here, we find almost identical gas exchange dynamics of *B. distachyon* stomata and can confirm that stomatal closing is faster than stomatal opening in *B. distachyon*, which was shown for many other grass species [44]. For *B. stacei* and *B. hybridum,* on the other hand, this aspect is more ambiguous, as the *k* and *Sl*_max_ values indicate faster opening rather than closing, but the lag times are shorter in the closing transitions (Table 4).

While we expected to find differences between polyploid *B. hybridum* and the two diploid species, we were surprised to find that *B. stacei* showed much lower physiological steady-state values in high light despite its close anatomical resemblance to *B. distachyon* stomata. Based solely on stomatal anatomical data, we are unable to explain this difference. We thus hypothesised that *B. stacei* might be either less responsive or adapted to intense light conditions than *B. distachyon* and *B. hybridum,* or it lacks a morphological, biochemical or biophysical component in its stomatal complexes. Generally, *B. stacei* was more likely to grow in shaded areas, whereas *B. distachyon* and *B. hybridum* were found in open, sun-exposed habitats, possibly due to adaptation of the latter to the generally more stressful dry and bright locations [31]. Additionally, a recent study investigating populations of *B. stacei* in a canyon area in Israel suggests a subspeciation of the taxon into a more arid- and more forest-adapted group [51]. Therefore, *B. stacei* used in our study might also belong to a more forest-adapted group and might not be well-suited to light-intensive conditions. Either genotypic analysis of our *B. stacei* population or comparison of stomatal anatomy and physiology of the three *B. stacei* populations identified would be required to test this.

The ultrastructure analysis of the graminoid stomata in the three *Brachypodium* species did not reveal differences between the three species but confirmed and further resolved aspects of grass stomatal morphology described previously [9, 49, 50, 52]. The cell wall thickness of stomatal cells is anisotropic and, in many areas, much thicker than average epidermal cell walls. This feature of cell wall structure is directly related to their ability to change shape in response to changes in turgor pressure and, thus, control the size of the stomatal opening [10, 14]. In our research, we found that GCs and SCs are symplastically isolated, which allows for reciprocal turgor regulation, as suggested before [14, 18]. In the analysed *Brachypodium* species, SCs maintain plasmodesmal connections to adjacent epidermal cells. Yet, if these plasmodesmata are transductive or blocked, and how they affect independent, reciprocal turgor regulation in SCs distinct from neighbouring pavement cells remains to be determined.

Finally, GCs must undergo reversible cell changes and thus possess unique cell wall material properties, which allow for repeated cycles of stomatal movement. The components that could influence such repeated movements are pectins [6, 53]. Recent work by Gkolemis et al. (2023) highlights the critical role of cell wall anisotropy in stomatal movement, demonstrating that the spatial organisation and mechanical properties of the cell wall matrix are essential for proper stomatal function [54]. In our work, we used antibodies against pectin epitopes with different degrees of methyl-esterification. It should be noted that different patterns of de-methyl-esterification may lead to opposing effects on cell wall mechanics [10, 55, 56]. De-methyl-esterification facilitates homogalacturonan cross-linking via Ca^2+^ and contributes to cell wall stiffening. In contrast, random de-methyl-esterification makes homogalacturonan susceptible to degradation by such enzymes (for example, polygalacturonase or pectate lyases), resulting in cell wall loosening [10, 55, 56]. In *Zea mays*, esterified/de-esterified pectic homogalacturonan dynamics favour cell wall expansion and deformation in elongating stomata [57, 58], with Giannoutsou et al. (2016) showing that these modifications are developmentally regulated in GCs and SCs. Gkolemis et al. (2023) further support this by showing that dynamic changes in pectin esterification states in *Zea mays* guard cells are crucial for cell wall flexibility during stomatal movement, reinforcing the importance of pectin remodelling in stomatal mechanics [54]. Our findings in *Brachypodium* extend this understanding, demonstrating that while pectin modifications are largely conserved across species, their specific distribution patterns may have functional consequences for stomatal performance.

Notably, we found pectins to be a highly abundant component of the stomata. Moreover, some of the analysed epitopes were present only in GCs (LM13) or highly polarised at specific locations like the transverse apical and basal walls of GCs (2F4). Interestingly, Giannoutsou et al. (2020) observed similar 2F4 polarisation of the signals in GCs mature stomata in *Vigna sinensis* [58]. While in *Zea mays*, the 2F4 epitope was present in all anticlinal cell walls of young stomata, with particularly strong localisation in transverse and lateral GCs walls. As stomata elongated, this epitope remained prominent in lateral and transverse GC walls, while in mature stomata, it became predominantly localised in the lateral walls of the central canal [58]. Strikingly, LM6 was the only antibody differentially distributed in stomatal complexes of the three *Brachypodium* species. This is particularly interesting in light of findings from Giannoutsou et al. (2016), who observed that LM6-labelled arabinans show developmental stage-specific and cell-type-specific localisation patterns in *Zea mays* stomatal complexes, being particularly abundant in guard mother cells and young GCs during early developmental stages. In our study, LM6 strongly stained SC walls in *B. distachyon* and (more weakly) in *B. hybridum*, but no staining was observed in SCs of *B. stacei*. The differences in LM6-labelled arabinan signals we observed in our study may be due to differential expression of genes related to arabinan biosynthesis in SCs. Alternatively, the copy number of these genes or gene families varies across species and could therefore affect arabinan abundance. Analysing the expression of these genes using cell- or tissue-type-specific transcriptome analysis in these three species may help to further elucidate this.

Ultimately, biomechanical changes to SCs due to absent or different cell wall components could explain the difference in stomatal opening, as differential SC stiffness likely interferes with their ability to biomechanically accommodate GC movement and thus stomatal opening. How and if arabinan abundance in SCs affects gas exchange capacity remains correlative in this study, as immunostainings are qualitative rather than quantitative. To demonstrate a causal and linear link between arabinan abundance and gas exchange capacity, SC-specific perturbations of arabinan deposition would be required. However, arabinans were previously suggested to be important for stomatal opening across a range of species, including the grass *Zea mays* and the grass relative *Commelina communis* [59, 60]. Nonetheless, a manifold of other leaf-specific or stomata-specific aspects like changes to the photosynthetic machinery in the mesophyll, differently sensitised or wired signalling modules in stomatal cells or cell wall components not assessed in this study could underlie the observed stomatal opening deficiency in *B. stacei*.

While this study provides insights into the structural and functional differences in stomatal anatomy and physiology among the *Brachypodium* species complex (genotypes: Bd21-3, ABR114 and ABR113), it is important to acknowledge that our findings are based on a single accession per species. Given that intraspecific variation in stomatal traits—such as size, density, and gas exchange kinetics—was documented in other grasses (e.g. [23], the observed interspecific differences may not fully represent the diversity within each species. For instance, environmental adaptation, ploidy-level variation, or genetic background could influence stomatal properties across different accessions of *B. distachyon*, *B. stacei*, or *B. hybridum*. Future studies incorporating multiple accessions per species would help determine whether the patterns reported here—such as the reduced gas exchange capacity in *B. stacei* or the differential arabinan localisation in SCs—are consistent across broader genetic diversity within a species or specific to the accessions studied. Nevertheless, our findings establish a foundational framework for comparative analyses in *Brachypodium* and highlight key anatomical and physiological traits that may underlie species-specific stomatal behaviour.

## Supplementary Information

Supplementary Material 1

Supplementary Material 2

Supplementary Material 3

Supplementary Material 4

Supplementary Material 5

Supplementary Material 6

Supplementary Material 7

Supplementary Material 8

Supplementary Material 9

Supplementary Material 10

Supplementary Material 11

Supplementary Material 12

Supplementary Material 13

Supplementary Material 14

Supplementary Material 15

Supplementary Material 16

## Data Availability

All data supporting the findings of this study are available within the paper or within its supplementary data. The R script used for calculations and visualisation in this study, as well as the LI-6800 files for physiological measurements and Excel tables containing measurements for stomatal anatomy and density, are available on Github under [https://github.com/raissig-lindner-lab/Berg-et-al_2025_Brachy-species-complex]. Further inquiries can be directed to the corresponding authors.

## Abbreviations

*A*: Carbon assimilation
AGP: Arabinogalactan proteins
BB: Blocking buffer
FB: Fluorescent brightener
GA: Glutaraldehyde
GCs: Guard cells
*g*_sw_: Stomatal conductance to water vapour
iWUE: Intrinsic water-use efficiency
PAR: Photosynthetically active radiation
PBS: Phosphate-buffered saline
PFA: Paraformaldehyde
PPFD: Photosynthetic photon flux density
SC: Subsidiary cells
SD: Stomatal density
TEM: Transmission electron microscopy
