# Supplementary material for: Stomatal anatomy and gas exchange dynamics in the Brachypodium distachyon complex
Source: BMC Plant Biol. 2025 Oct 10;25:1360. doi: 10.1186/s12870-025-07312-0 (PMC12512691; doi:10.1186/s12870-025-07312-0)
Supplement: Supplementary file 1 — Supplementary Material 1 [file 12870_2025_7312_MOESM1_ESM.pdf]

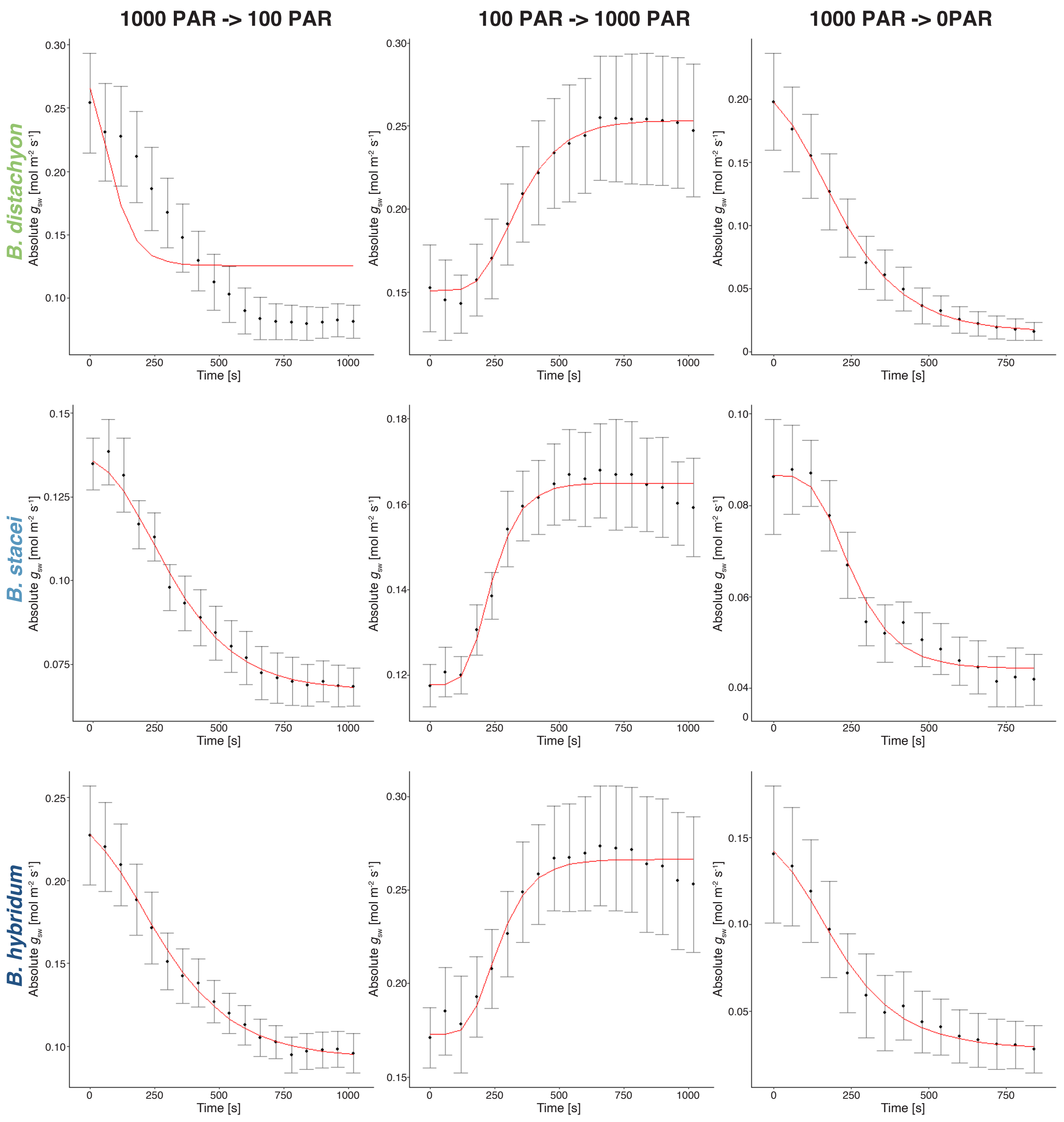

Fig. S1. Licornetics plots of stomatal kinetics model. Shown are different light transitions from left to right; high light to low light, low light to high light, and high light to no light. All three species are shown; *B. distachyon* (first row), *B. stacei* (second row) and *B. hybridum* (third row). Modelling was done on the data shown in Figure 2, n = 5 individuals per species.

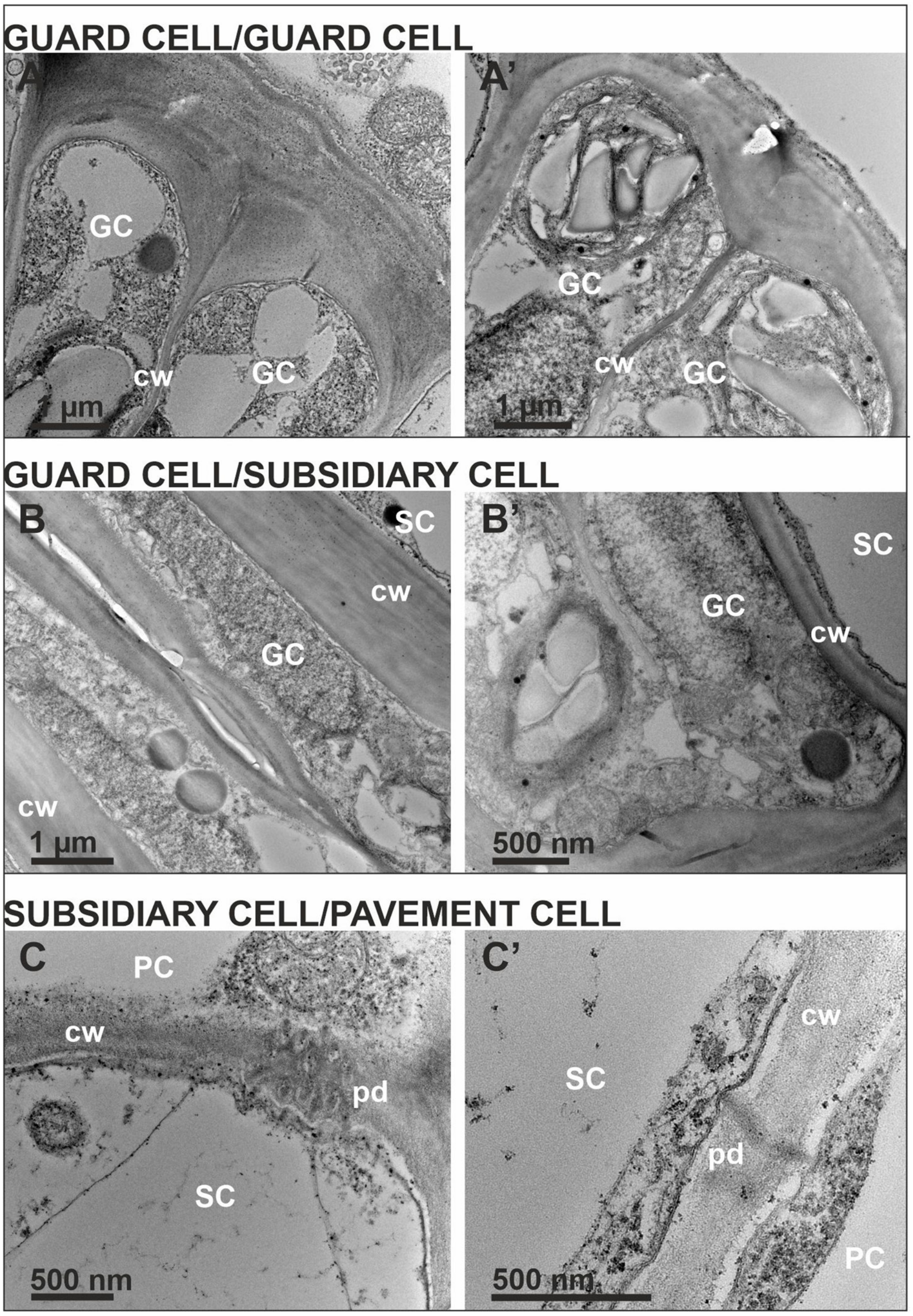

Fig. S2. Electron micrographs of stomatal cells in *B. distachyon*, showing plasmodesmata (PD) between:

Guard cell and guard cell (A, A')

Guard cell and subsidiary cell (B, B')

Subsidiary cell and pavement cell (C, C').

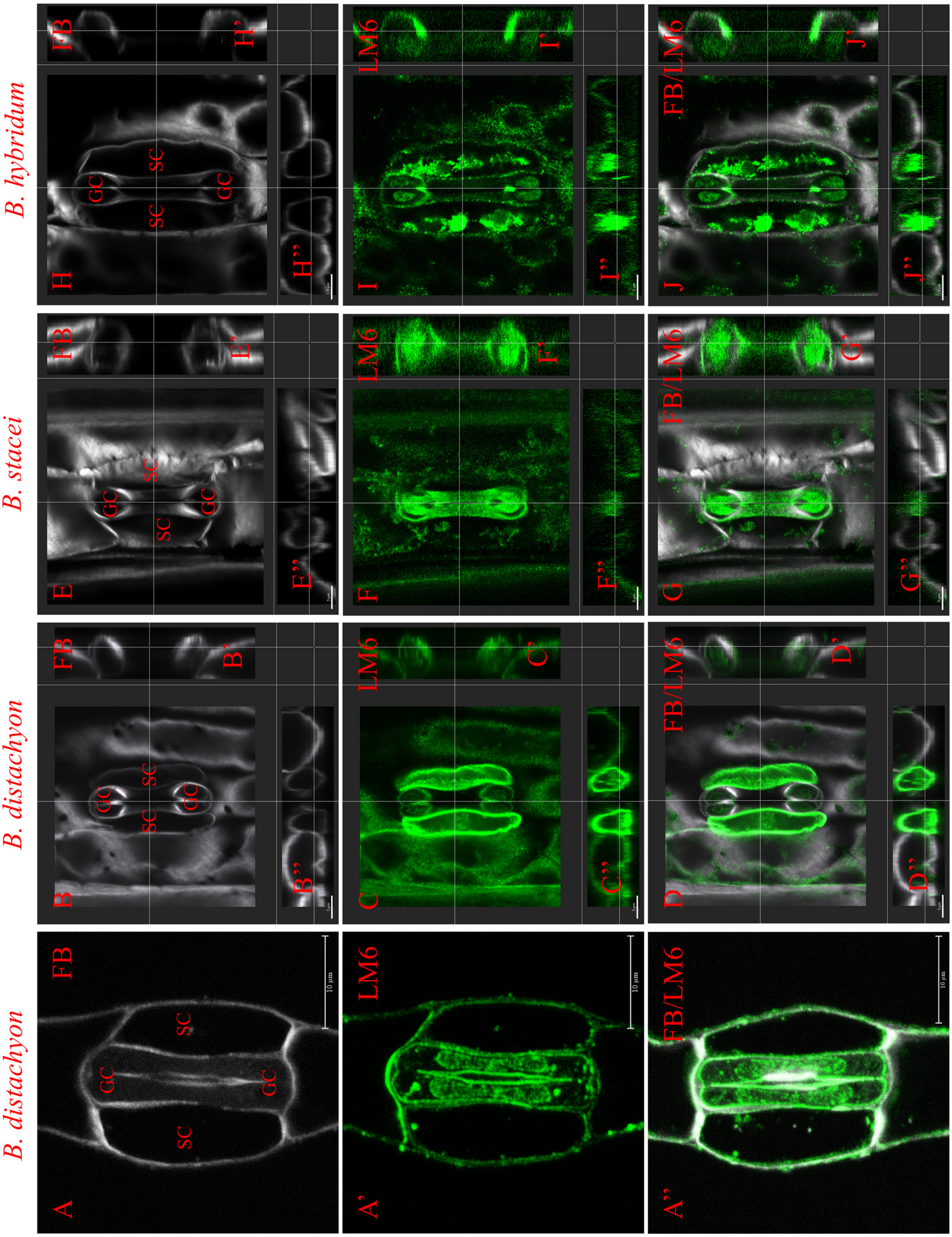

Fig. S3. Immunolocalization of pectins (LM6 epitope) in the stomata of the *Brachypodium distachyon* complex.

A–A'': Histological sections of *B. distachyon*

B–D'': Whole-mount of *B. distachyon*

E–G'': Whole-mount of *B. stacei*

H–J'': Whole-mount of *B. hybridum*.

FB: Fluorescent brightener. Scale bars: 5 µm.

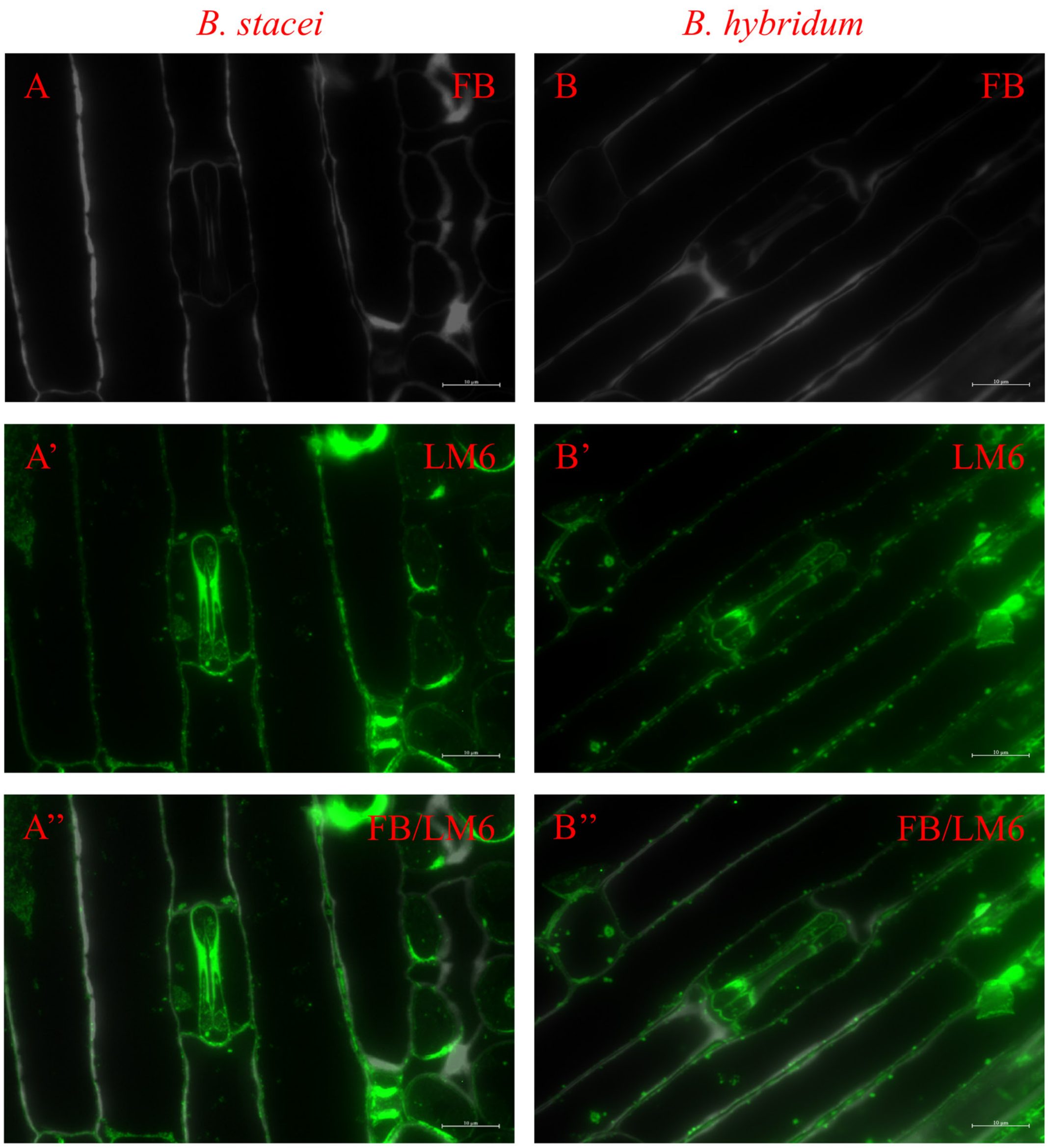

Fig. S4. Immunolocalization of pectins (LM6 epitope) in the stomata of the *Brachypodium distachyon* complex.

A–A'': Histological sections of *B. stacei*

B–B'': Histological sections of *B. hybridum*.

FB: Fluorescent brightener. Scale bars: 5 μm.

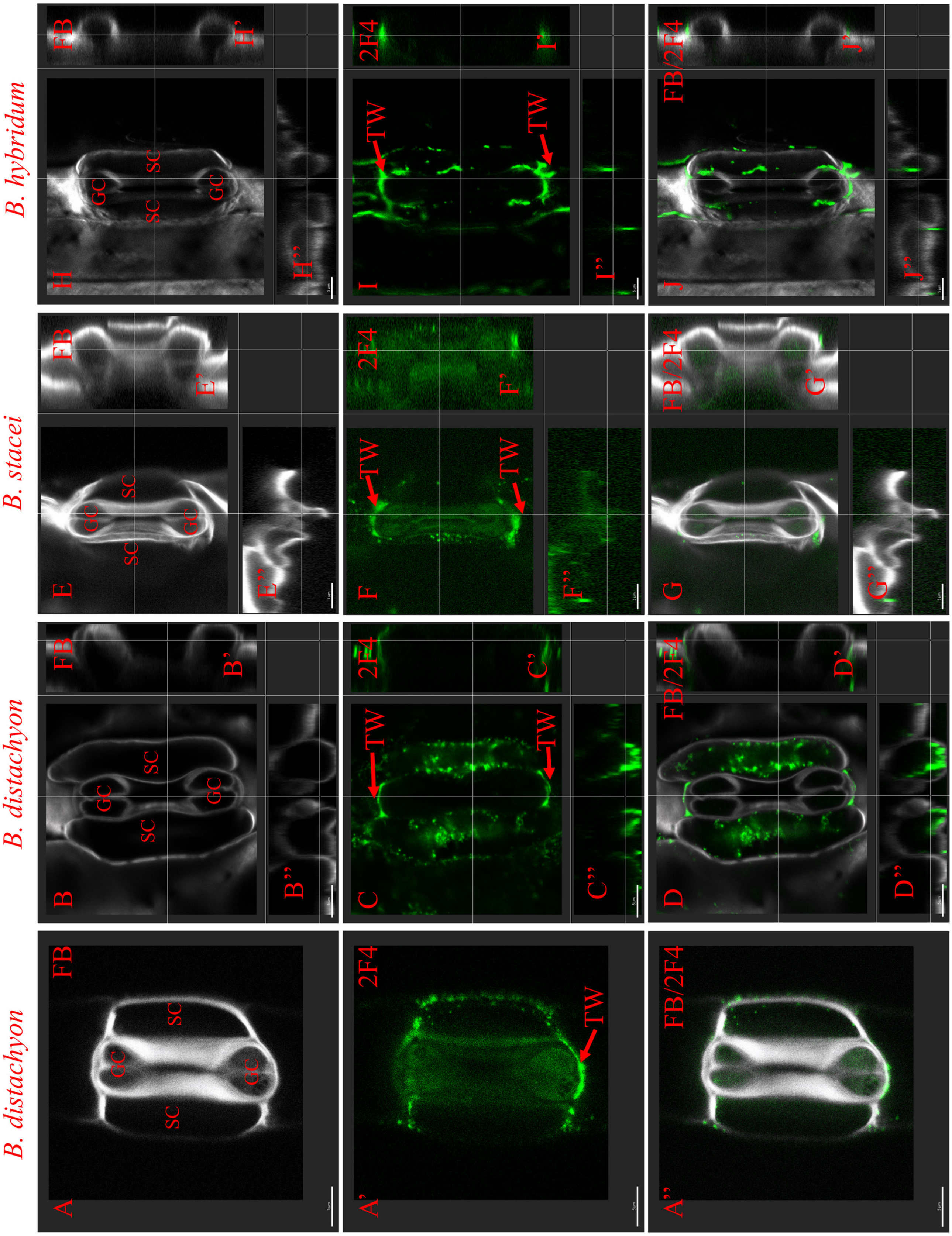

Fig. S5. Immunolocalization of non-esterified Ca^{2+}-cross-linked homogalacturonan (2F4 epitope) in the stomata of the *Brachypodium distachyon* complex.

A–A'': Histological sections of *B. distachyon*

B–D'': Whole-mount of *B. distachyon*

E–G'': Whole-mount of *B. stacei*

H–J'': Whole-mount of *B. hybridum*.

FB: Fluorescent brightener; TW: transverse wall. Scale bars: 5 μm.

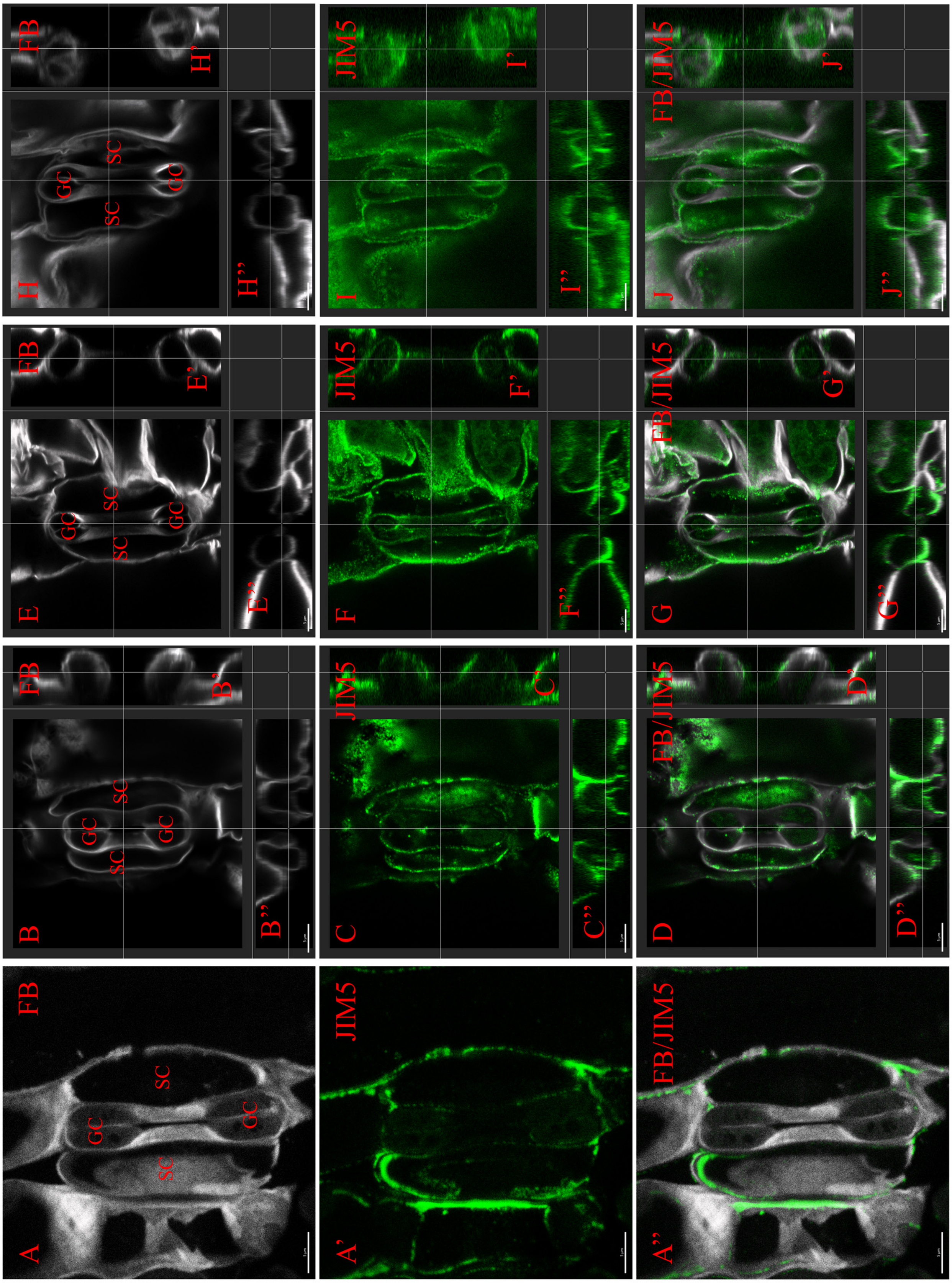

Fig. S6. Immunolocalization of unesterified homogalacturonan (JIM5 epitope) in the stomata of the *Brachypodium distachyon* complex.

A–A'': Histological sections of *B. distachyon*

B–D'': Whole-mount of *B. distachyon*

E–G'': Whole-mount of *B. stacei*

H–J'': Whole-mount of *B. hybridum*.

FB: Fluorescent brightener. Scale bars: 5 μm.

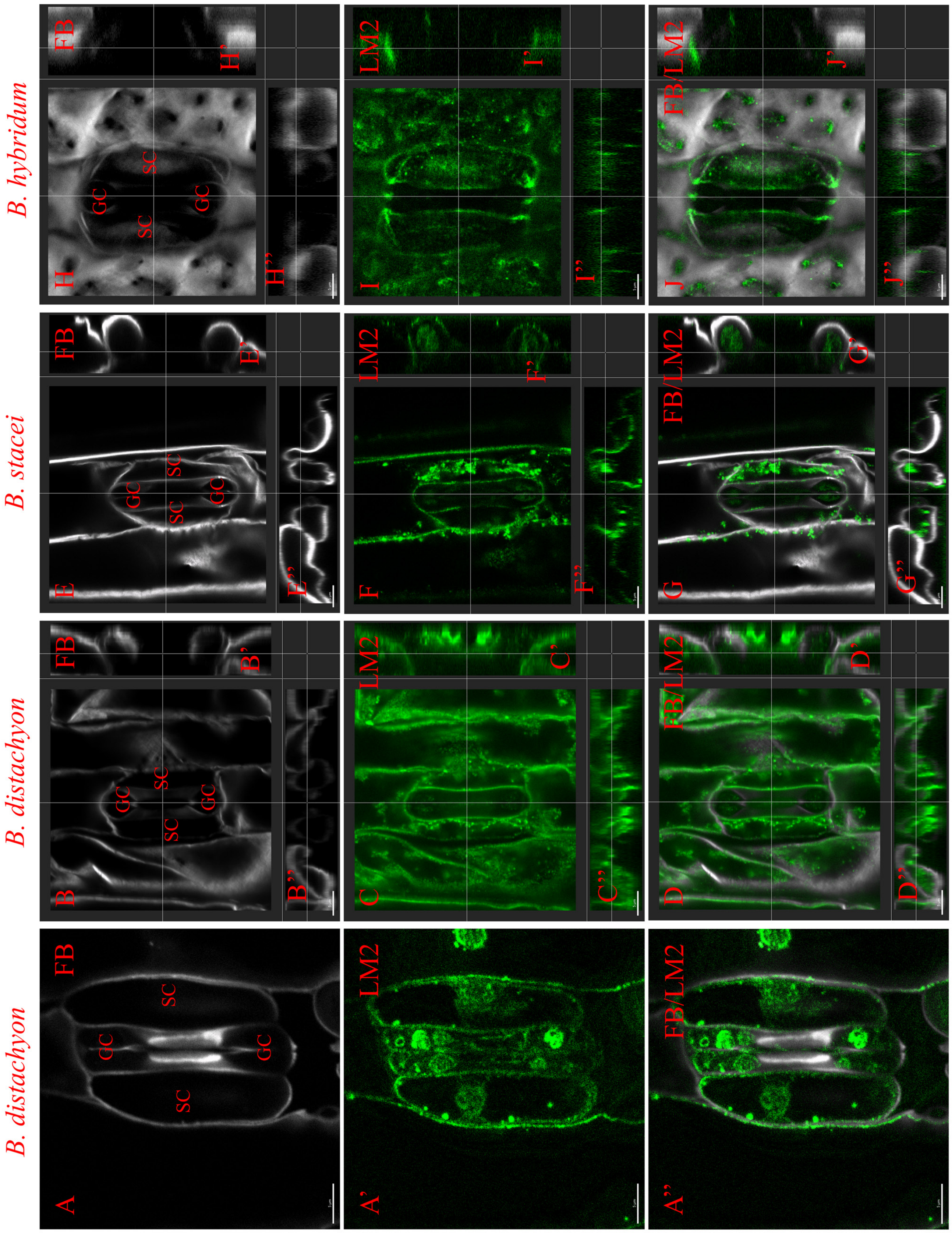

Fig. S7. Immunolocalization of arabinogalactan protein (LM2 epitope) in the stomata of the *Brachypodium distachyon* complex.

A–A'': Histological sections of *B. distachyon*

B–D'': Whole-mount of *B. distachyon*

E–G'': Whole-mount of *B. stacei*

H–J'': Whole-mount of *B. hybridum*.

FB: Fluorescent brightener. Scale bars: 5 μm.

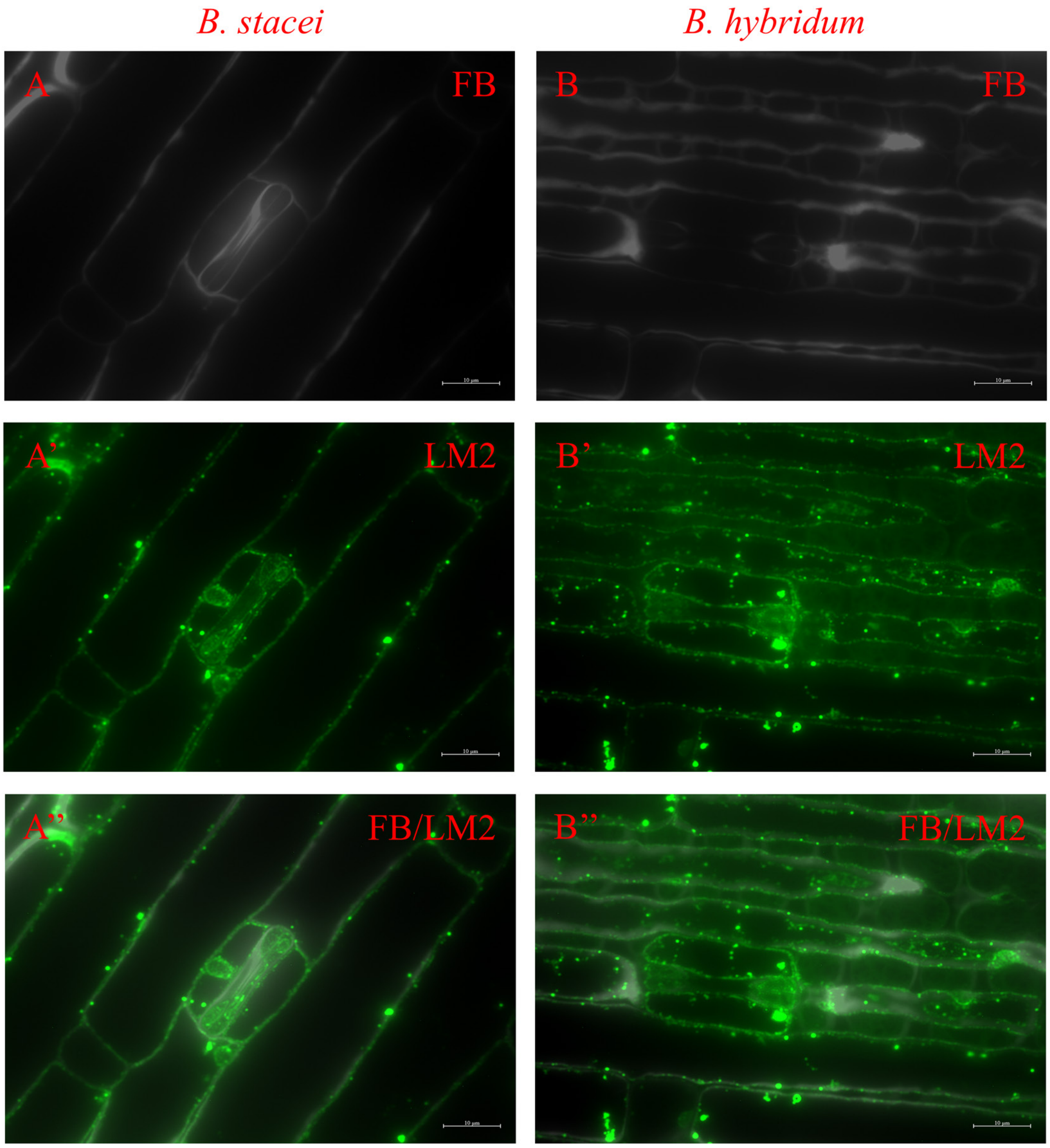

Fig. S8. Immunolocalization of arabinogalactan protein (LM2 epitope) in the stomata of the *Brachypodium distachyon* complex.

A–A'': Histological sections of *B. stacei*

B–B'': Histological sections of *B. hybridum*

FB: Fluorescent brightener. Scale bars: 5 μm.

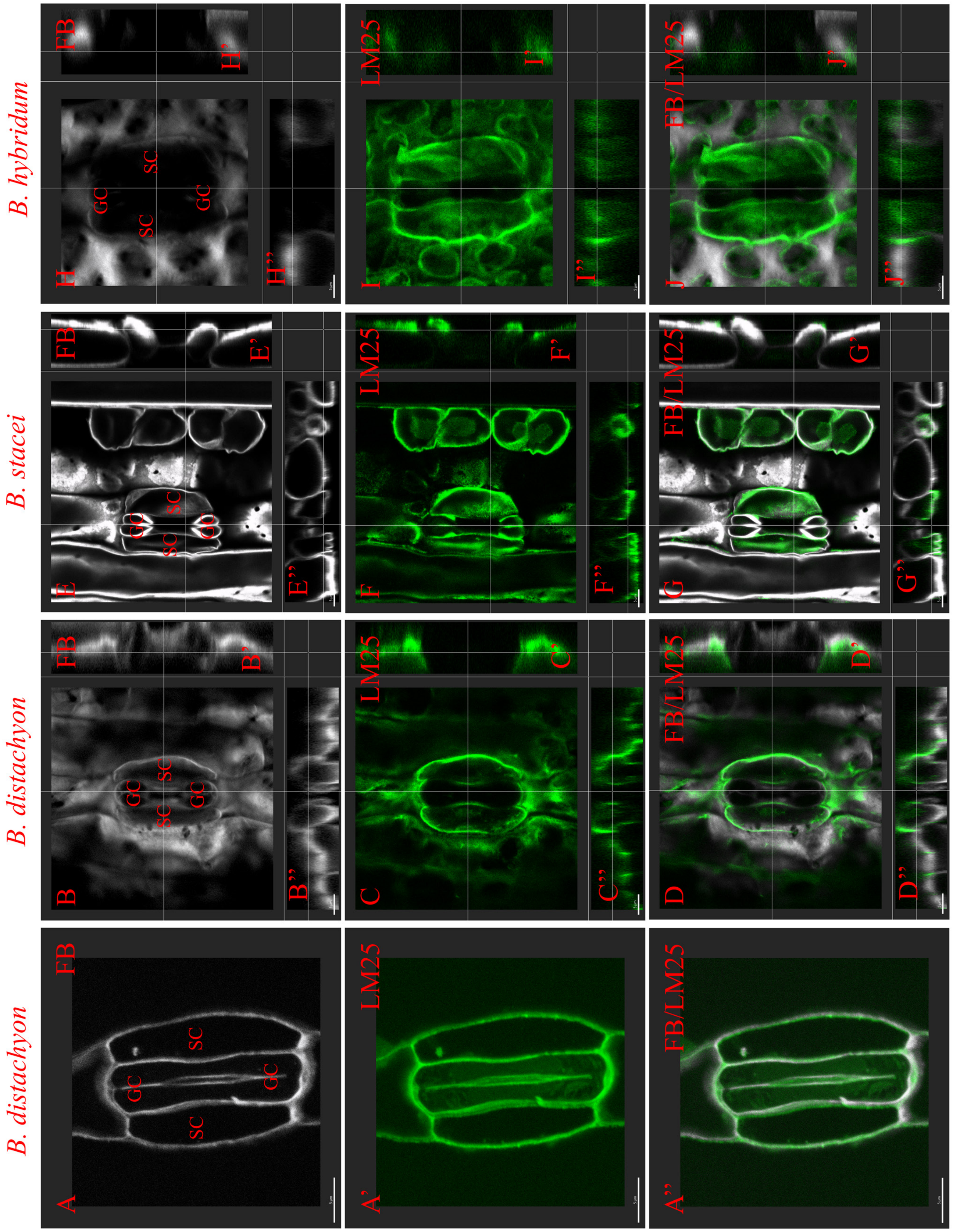

Fig. S9. Immunolocalization of xyloglucan (LM25 epitope) in the stomata of the *Brachypodium distachyon* complex.

A–A'': Histological sections of *B. distachyon*

B–D'': Whole-mount of *B. distachyon*

E–G'': Whole-mount of *B. stacei*

H–J'': Whole-mount of *B. hybridum*.

FB: Fluorescent brightener. Scale bars: 5 μm.

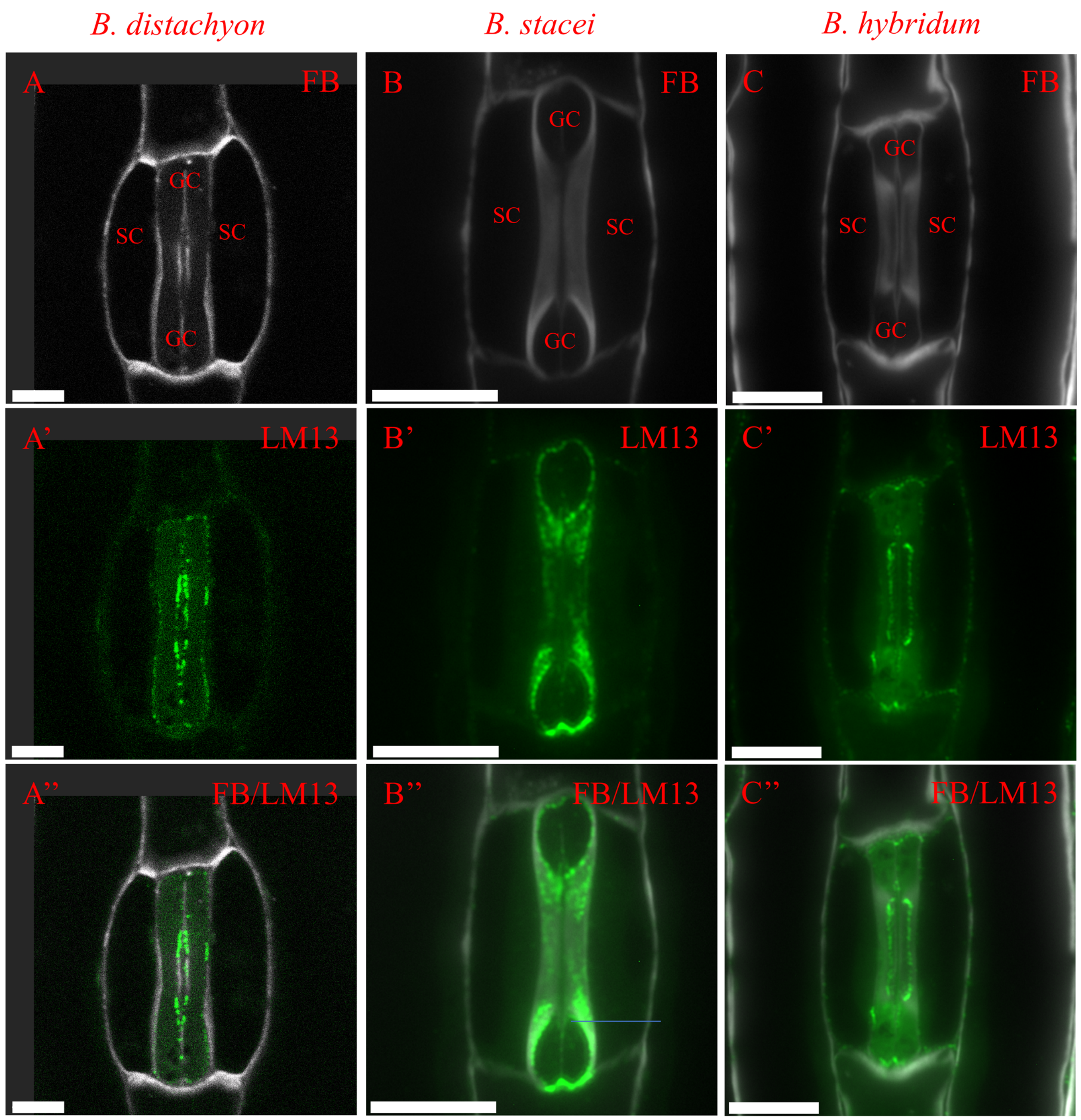

Fig. S10. Immunolocalization of (1,5)-α-L-arabinan (LM13 epitope) in the stomata of the *Brachypodium distachyon* complex.

A–A’’: Histological sections of *B. distachyon*

B–B’’: Histological sections of *B. stacei*

C–C’’: Histological sections of *B. hybridum*.

FB: Fluorescent brightener. Scale bars: 5 μm.

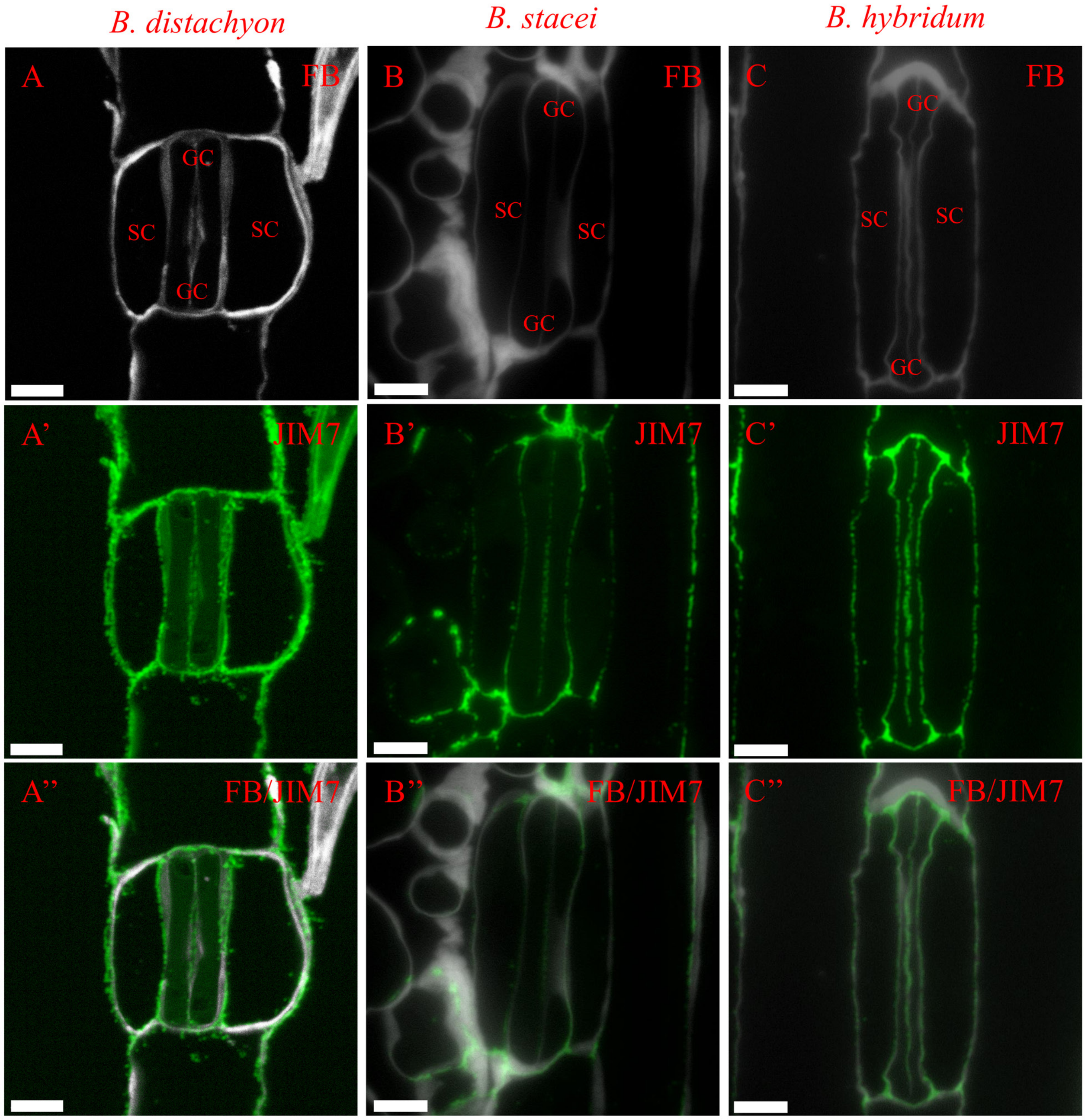

Fig. S11. Immunolocalization of methylesterified homogalacturonan (JIM7 epitope) in the stomata of the *Brachypodium distachyon* complex.

A–A'': Histological sections of *B. distachyon*

B–B'': Histological sections of *B. stacei*

C–C'': Histological sections of *B. hybridum*.

FB: Fluorescent brightener. Scale bars: 5 µm.

Video S1: Z-stacks of *Brachypodium distachyon* (1SA), *B. stacei* (1SB), and *B. hybridum* (1SC) stomata after immunocytochemistry with LM6. Results show only the antibody signal.

Video S2: Z-stacks of *B. distachyon* (2SA), *B. stacei* (2SB), and *B. hybridum* (2SC) stomata after immunocytochemistry with 2F4. Results show only the antibody signal.

Video S3: Z-stacks of *B. distachyon* (3SA), *B. stacei* (3SB), and *B. hybridum* (3SC) stomata after immunocytochemistry with JIM5. Results show only the antibody signal.

Video S4: Z-stacks of *B. distachyon* (4SA), *B. stacei* (4SB), and *B. hybridum* (4SC) stomata after immunocytochemistry with LM2. Results show only the antibody signal.

Video S5: Z-stacks of *B. distachyon* (5SA), *B. stacei* (5SB), and *B. hybridum* (5SC) stomata after immunocytochemistry with LM25. Results show only the antibody signal.
